# Gigavoxel-scale multiple-scattering-aware lensless holotomography

**DOI:** 10.1038/s41377-026-02416-0

**Published:** 2026-09-16

**Authors:** Mikołaj Rogalski, Julianna Winnik, Julia Dudek, Piotr Arcab, Emilia Wdowiak, Paweł Matryba, Marzena Stefaniuk, Piotr Zdańkowski, Maciej Trusiak

**Affiliations:** 1 Warsaw University of Technology, Institute of Micromechanics and Photonics, Faculty of Mechatronics, Warsaw, Poland; 2 Laboratory of Neurobiology, Nencki Institute of Experimental Biology of Polish Academy of Sciences, Warsaw, Poland; 3 Department of Immunology, Medical University of Warsaw, Warsaw, Poland

**Keywords:** Imaging and sensing, Optical metrology, Phase-contrast microscopy, Interference microscopy, Biophotonics

## Abstract

Holotomography (HT) has revolutionized quantitative label-free 3D imaging, yet conventional lens-based implementations are fundamentally constrained in field-of-view (FOV) and imaging depth, limiting their utility for critical high-throughput applications in material and life sciences. Lensless HT (LHT) offers a promising alternative for large-volume examination, however existing approaches fail to accurately reconstruct highly scattering samples over extended depths, which remains a critical challenge in optical imaging field. Here, we introduce a gigavoxel-scale, multiple-scattering-aware LHT with a large FOV (surpassing 0.6 cm^2^), millimeter-scale axial range, and pixel level (~2.4 µm) resolution. Our approach leverages a multi-wavelength, oblique-illumination hologram reconstruction and a robust, automatic illumination angle calibration, which are necessary for precise large-volume 3D holographic reconstruction. Moreover, we propose optimization-driven multi-slice tomographic framework to accurately capture multiple scattering effects outperforming first order Born/Rytov-based inversions. To rigorously validate our method, we reconstruct bespoke multi-layer two-photon polymerized test structure over a 1.7 mm imaging depth and 25 mm^2^ FOV, yielding an unprecedented 3D space-bandwidth product exceeding a gigavoxel level. Furthermore, we demonstrate for the first time on-chip label-free imaging of entire 500-µm-thick tissue slice of optically cleared mouse brain. With the proposed method, we aim to unlock powerful new capabilities for large-scale, quantitative, label-free 3D imaging across biomedicine, neuroscience, material sciences and beyond.

## Introduction

Three-dimensional (3D) label-free optical imaging is a transformative tool in modern microscopy, enabling the visualization of biological and technical specimens with high spatial resolution while preserving their native volumetric structure^1^. Among the most powerful techniques, optical diffraction tomography (ODT)^2,3^ has emerged as indispensable method for quantitative phase imaging, providing deep insights into the internal organization of transparent samples without the need for staining. ODT can be mainly realized via interferometric coding as holotomography (HT)^4^ or non-interferometric coding as intensity diffraction tomography^5^ or Fourier ptychographic tomography^6,7^. These 3D imaging methods have significantly advanced fields such as biomedicine, materials science, and neuroscience, allowing for the study of cellular and tissue architecture, engineered microstructures, and complex scattering media^1–7^.

Despite their success, conventional implementations remain fundamentally constrained by the optical limitations of lens-based systems, where the trade-off between numerical aperture, field-of-view (FOV), and imaging depth imposes severe restrictions on practical applications. High-resolution, high-quality imaging is inherently limited to small volumes, while attempts to increase the accessible FOV often come at the cost of lower resolution^8^, reduction of the illumination angles and spatial fidelity^9^ or laborious and erroneous scanning and stitching^10,11^.

Lensless holotomography (LHT) has recently emerged as a promising alternative, leveraging on-chip defocused intensity detection and digital holographic wavefield processing to enable tomographic imaging over large volumes without the need for bulky optical elements. By circumventing the constraints of conventional lenses, LHT holds the potential to dramatically expand the scale and versatility of tomographic imaging. LHT is a relatively new field, still in its proof-of-concept phase, with only a modest number of studies addressing its inherent challenges.

In a pioneering 2011 paper^12^, Ozcan’s group employed orthogonal illumination via a mechanically scanned optical fiber imaging scattering biological samples (*C. elegans*) up to ~50 µm thick. The use of an LED light source introduced coherence limitations on information content and resolution, especially for objects placed far from the sensor^13^. The 2D reconstruction lacked twin-image removal, while the 3D reconstruction did not account for multiple scattering. A portable setup based on this method demonstrated impressive results^14^ but exhibited reduced resolution. Ozcan’s group also showed a volumetric 200 µm thick mouse brain slice image^15^, however the 3D effect was achieved via z-stacking and not through rigorous Fourier diffraction theorem (ODT).

Zuo et al.^16^ used a color LED matrix for illumination and aimed at wide-field volumetric imaging. While enabling a large FOV (24 mm²), imaging depth remained limited to ~50 µm, likely due to the small number of angles and restricted coherence of the LED source. Berdeu et al.^17,18^ developed an LHT platform based on a rotatable robotic arm and incorporated diffraction effects by applying the Fourier diffraction theorem inversion under the first-order Born approximation. However, additional image transformations (e.g., z-scaling, transverse shifts) - prone to illumination angle errors - were required, reducing imaging fidelity. The reliance on single-hologram noniterative reconstructions^17^ led to strong twin-image artifacts, necessitating heavy regularization and sparsity priors that generally decrease the accuracy and resolution of 3D reconstructions of thick and dense multiple-scattering bio-structures^17–19^. Luo et al.^20^ introduced a scan-free LHT system that uses only four laser diodes yielding a limited volume of interest (3.4 × 2.3 × 0.3 mm³) with a highly total-variation regularized (applicable to piecewise-constant samples) first-order Born reconstruction.

Recent developments in 2024 further highlight the field’s rapid expansion. Zhou et al.^21^ proposed an LED-based setup incorporating a deconvolution-driven tomographic reconstruction algorithm with a limited imaging volume (930 × 930 × 200 µm³). Qin et al.^22^ demonstrated a single-shot portable LED-matrix-based LHT system with limited imaging depth to 50 µm. Zuo’s group^22^ further advanced LHT with a motion-free setup employing wavelength scanning. Despite using a broad spectral range (430–1200 nm), posing a risk of dispersion-induced errors, axial 3D Fourier space coverage remained limited.

Although these methods have demonstrated the feasibility of large scale label-free tomographic imaging on a lensless platform, they predominantly rely on direct inversion under the first-order Born or Rytov approximation^2^, which is inadequate for multiple scattering in dense or thick samples^3^. These proof-of-concept approaches are typically tailored to specific cases involving sparse or weakly scattering objects, such as microbeads and thin cellular structures. In addition, LHT systems apply hologram recording in a defocused plane, which poses a major challenge when applying tilted illumination. Despite its conceptual appeal, LHT has yet to provide a comprehensive solution that delivers high-fidelity, robust, large-volume imaging with versatility required for practical deployment.

Addressing these crucial problems, we introduce a gigavoxel-scale multiple-scattering-aware LHT approach for precise, large-FOV and deep millimeter-scale 3D label-free phase (refraction) and amplitude (absorption) imaging. In our approach we leverage a set of novel methods: (1) robust, automated sub-degree-precision illumination angle calibration strategy, (2) 2D complex fields reconstruction from multi-wavelength inclined Gabor holograms using specialized oblique-illumination diffraction computation and (3) a reliable, optimization-driven, computationally efficient, tomographic reconstruction framework with multiple scattering correction. We demonstrate the effectiveness of our numerically enhanced LHT framework through the successful 3D imaging of inclined phase test target, two-photon-printed bespoke volumetric calibration structure and challenging mouse brain tissue slice of unprecedented 500 µm thickness.

## Results

### Holotomographic data acquisition and calibration

**Fig. 1:**
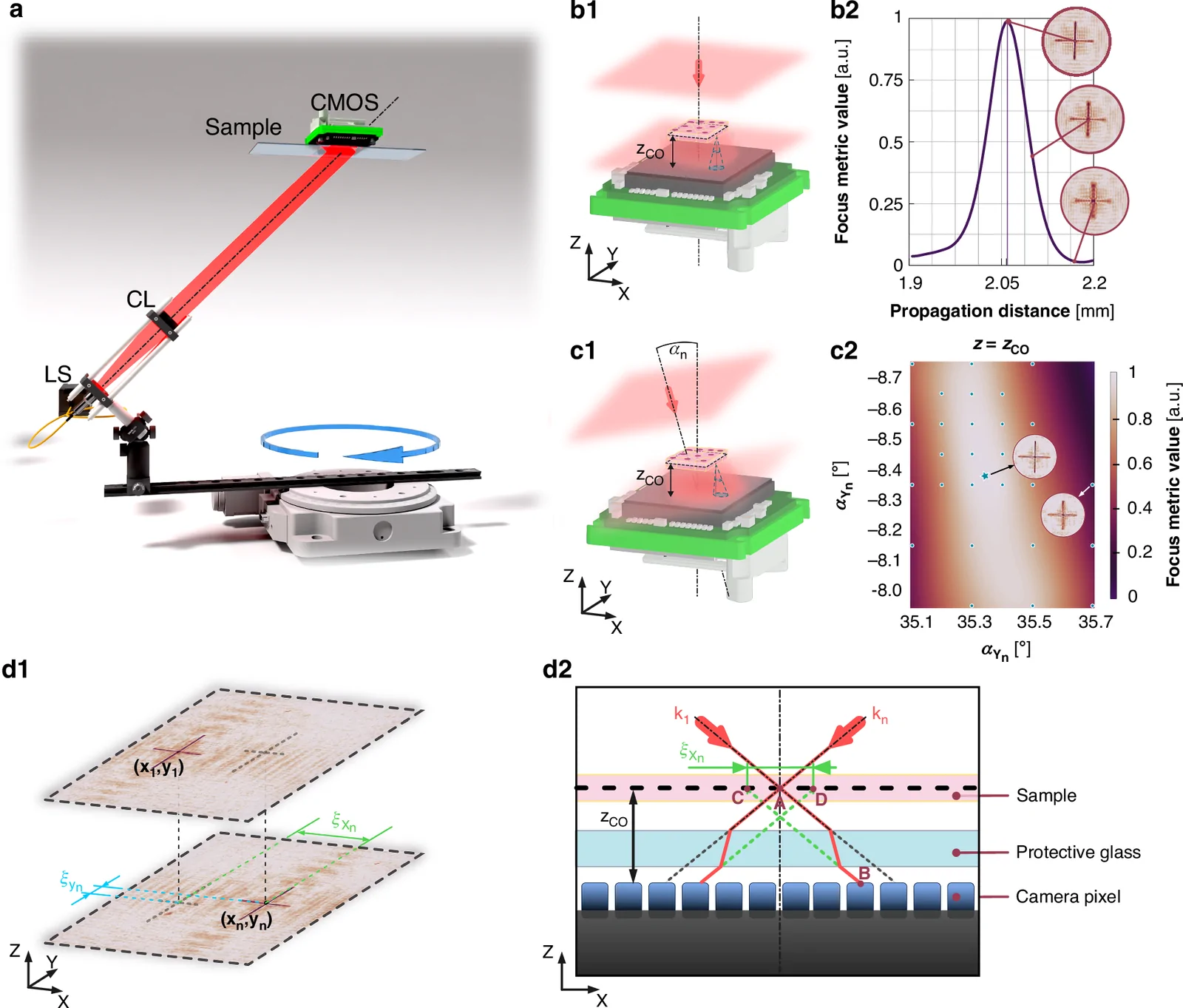
Lensless holotomograph design and calibration principle. **a** Schematic illustration of the lensless gigapixel-scale quasi-isotropic holotomography setup. A laser source (LS) emits (sequentially with ${\lambda }_{1}=540$ and ${\lambda }_{2}=640$ nm) a coherent beam that is collimated by a collimating lens (CL) to illuminate the sample at an oblique angle (approx. 45°). LS is mounted on a precision rotation stage, enabling full angular diversity for conical shape tomographic illumination pattern. A high-resolution CMOS sensor captures the diffracted light without the use of imaging lenses, enabling lensless gigavoxel-scale reconstructions. **b**1 System configuration for acquiring the on-axis calibration-only hologram. **b**2 DarkFocus metric values computed for the CO across various propagation distances under normal illumination $({\alpha =0^{\circ}})$. **c**1 System configuration for oblique illumination. **c**2 DarkFocus metric evaluated at different candidate illumination angles, with the axial distance fixed at ${z}_{{co}}$; blue dots represent the calculated metric values, color-coded image represents the 2D polynomial fitted to calculated values and blue star represents the found illumination angle (location of the maximum of the polynomial). (**d**1) Lateral misalignment between reconstructions of the CO for 1^st^ and n^th^ illumination angles, quantified as ${\xi }_{{x}_{n}}$, ${\xi }_{{y}_{n}}$. **d**2 Illustration of the Snell effect influence on the system: an object at position A illuminated with wavevector ${k}_{1}$ appears at position B on the camera sensor due to refraction by the cover glass. Reconstruction of 1^st^ hologram under ${k}_{1}$ yields a shifted image CO at point C, while reconstruction of n^th^ hologram under ${k}_{n}$ leads to CO image at D, offset by ${\xi }_{{x}_{n}}$ from C

Figure 1a illustrates the proposed experimental setup consisting of a board-level camera, the measured sample and inclined (approx. 45°, to be adjusted depending on the measurement case) illumination via collimated fibre-coupled supercontinuum laser with tunable wavelength. The output of the fibre is mounted on a motorized rotation stage, enabling precise angular scanning across a full 360° range. At each angular position (in total 180 projections with 2° increment), two inclined Gabor holograms are recorded – one using ${\lambda }_{1}=540$ nm and the other ${\lambda }_{2}=640$ nm illumination wavelength – for efficient phase retrieval.

Unlike lens-based HT systems limited by microscope objective’s parameters, lensless configurations can reconstruct optical fields over substantially larger etendue (Supplementary Note 1). However, achieving high reconstruction accuracy over such volumes requires precise knowledge of the illumination angle (${\alpha }_{n}$) for each n^th^ collected hologram (defocused image). Any, even arcminute, angular inaccuracies lead to transverse misalignments of the propagated fields and distortions of retrieved projections, severely degrading final 3D reconstruction quality. For instance, propagating an optical field over a 1 mm distance at 40° illumination generates a 1.2 µm transverse error for just a 0.1° angle deviation.

To address this challenge, we developed SHARP – Snell-corrected Holographic Angle Recovery Protocol – a three-step lensless holotomography calibration procedure, Fig. 1. SHARP is designed for accurate estimation of the sample axial location and all illumination angles, to obtain distortion-free full-volume reconstructions, and additional correction of transverse misalignments caused by both residual angular deviations and the Snell effect due to refractive index mismatch between air, sample and the camera glass cover. SHARP requires acquiring a single additional hologram under on-axis illumination (Fig. 1b1) and the presence of a calibration object (CO) within the imaging volume. The CO is defined as a small (10–100 µm diameter), thin (<10 µm) structure that exhibits high spatial-frequency content and is spatially isolated from other features in the sample, e.g., a dust particle or a native object feature.

In the first step of the proposed method, the on-axis hologram is numerically propagated to multiple axial planes with the use of angular spectrum method^23–25^ (Fig. 1b1). At each plane, we compute the DarkFocus (variance of computational darkfield gradient) sharpness metric^26^ to localize the CO plane and determine its axial position ${z}_{{co}}$ relative to the camera (Fig. 1b2). In the second step, we analyze each n^th^ hologram acquired under oblique illumination (Fig. 1c1) to estimate the corresponding x and y illumination angle components: ${\alpha }_{{x}_{n}}$ and ${\alpha }_{{y}_{n}}$. For each dataset, the CO sharpness is evaluated using the DarkFocus metric applied to the holograms propagated to the previously identified ${z}_{{co}}$ plane, across a range of candidate illumination angles centered around the nominal values obtained from the acquisition system (blue points in Fig. 1c2). During this step, holograms are propagated using precise off-axis diffraction numerical modelling method, described in detail in Supplementary Note 3. The algorithm supports accurate, aliasing-free long-distance tilted-direction propagation under coarse sampling and a limited field of view (area around CO), precisely the regime in which SHARP operates. The final illumination angle is determined by locating the maximum of a second-order polynomial fit to the computed sharpness metric values.

Ideally, numerically propagating each hologram to the CO plane using the corresponding calibrated angle should yield reconstructions where the CO appears in-focus at the same transverse location. In practice, small residual angular errors and the refractive shift (due to the Snell effect) – caused by the sample glass slide, coverslip and protective cover glass on top the camera sensor – introduce transverse displacements, visualized in Fig. 1d. Therefore, in the third step, we compute the residual transverse shift (${{{\xi }}}_{{{{x}}}_{{{n}}}}$, ${{{\xi }}}_{{{{y}}}_{{{n}}}}$) between the CO reconstructions obtained under 1^st^ and n^th^ illumination angles using a cross-correlation approach (Fig. 1d1)^27^. This compensates for cumulative misalignments and ensures that the CO is correctly registered across all illumination angles. For the planes located far from the CO plane, residual angular calibration errors can still introduce transverse misalignments, potentially degrading lateral resolution. This effect can be mitigated by repeating the calibration procedure for additional axial planes. However, it is important to note that for all datasets presented in this work, no significant resolution loss was observed across volumes up to 10 × 5 × 2 mm³.

### Lensless multiple-scattering-aware tomographic large-volume reconstruction

Following the SHARP calibration procedure, the collected holograms ${I}_{{\lambda }_{1}}(x,y)$ and ${I}_{{\lambda }_{2}}(x,y)$ undergo preprocessing – they are shifted by *ξ*_*x*_ and *ξ*_y_ to correct for transverse misalignments and normalized by dividing them by their mean value. Next, the complex amplitudes in the camera plane, ${C}_{n}(x,y)$, are retrieved for each n^th^ illumination angle. For this, we use modified off-axis multi-wavelength Gerchberg–Saxton^16,28,29^ (GS) phase retrieval algorithm, tailored for volumetric sample imaging with oblique illumination (Algorithm S1 in Supplementary Note 4).

For efficient large-volume gigavoxel-scale robust 3D reconstruction, we propose a novel algorithm called Scattering-Optimized Lensless Volume Estimator – SOLVE. Our method effectively captures the multiple scattering effects that have long hindered LHT’s application to dense, axially extended complex samples. Firstly, an initial estimate of the 3D complex amplitude distribution is obtained using a modified filtered backpropagation (FB) algorithm^30^ (Algorithm S2 in Supplementary Note 5) for the user defined range of propagation distances and the axial sampling. The final 3D distribution (*U*) is then refined using the proposed iterative reconstruction algorithm.

Each iteration of the SOLVE algorithm (Algorithm S3 in Supplementary Note 6) begins with filtering the 3D complex volume *U* by applying a physical constraint that the normalized object amplitude must be less than or equal to 1 – meaning the object can absorb but not generate light. This step improves the convergence and stability of the reconstruction process. Next, for each illumination angle n, the multi-plane volume *U* is propagated to the camera plane using a multi-slice beam propagation method (BPM) model^31^. The propagation between individual slices of *U* is realized with the use of specialized off-axis shift-preserving AS method^32^, while propagation between last slice of *U* and camera is realized with shift-suppressed AS. Both AS variants are tailored to specific LHT conditions and are thoroughly described and motivated in Supplementary Note 3, offering additional insights in comparison to previous works^22,33^. The difference between the estimated field at the camera and the measured complex amplitude (retrieved from oblique GS) is then calculated. After computing a full set of differences, a 3D correction volume ΔU is reconstructed using the FB method. This ΔU is subtracted from the current *U* with an empirical learning rate, resulting in the updated estimate.

### Experimental verification

**Fig. 2:**
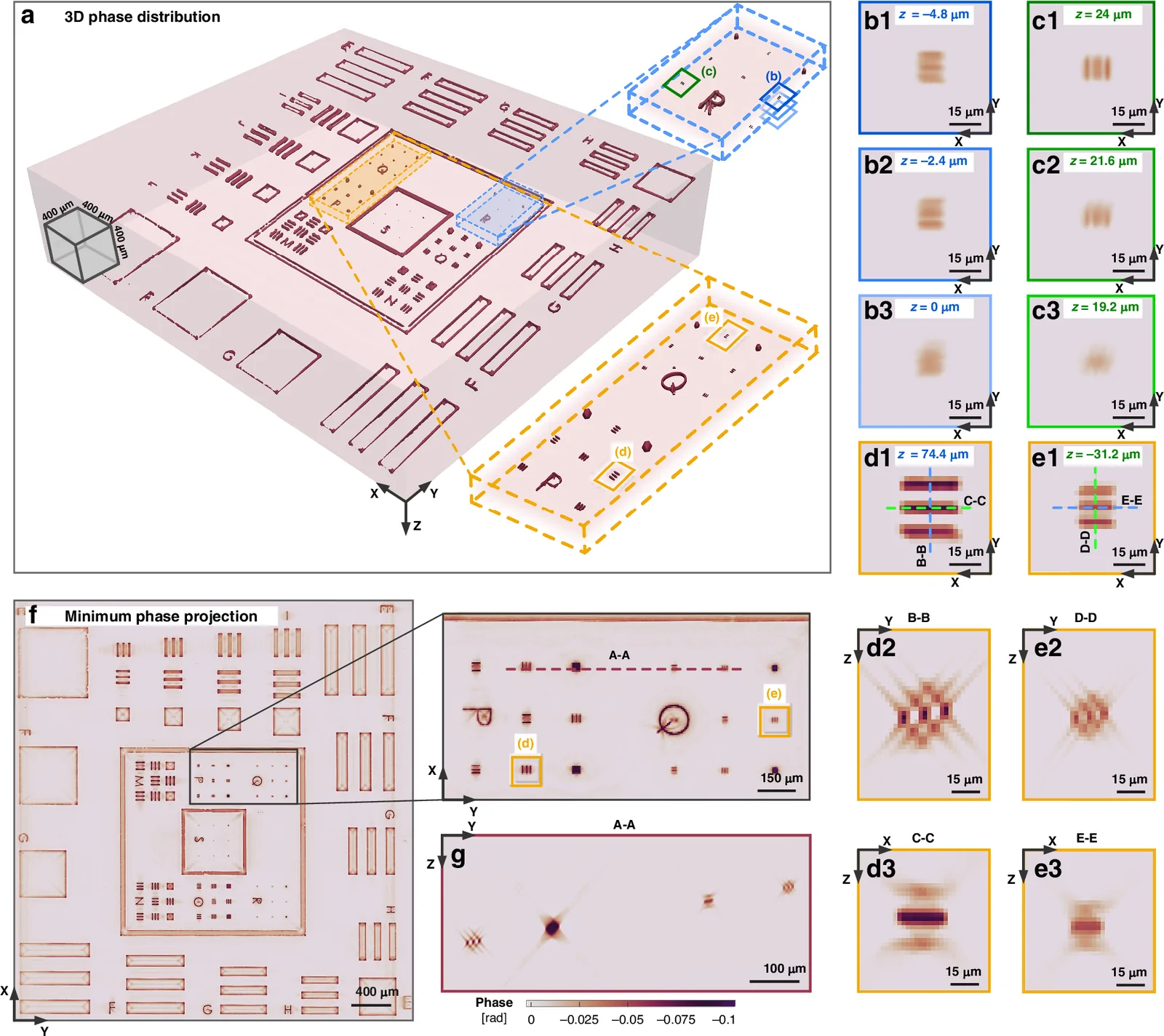
3D phase reconstruction of a resolution target. A custom phase resolution target (etched 125 nm features on Borofloat 33 glass) was imaged at a tilt of 25°. **a** 3D rendering of the reconstructed phase distribution. **b**1–**b**3 Transverse phase slices of 3 µm-wide features (group R) along x axis, in focus **b**1 and at different axial planes **b**2, **b**3. **c**1–**c**3 Transverse phase slices of 3 µm-wide features (group R) along y axis, in focus **c**1 and at different axial planes **c**2, **c**3. **d**1–**d**3 Transverse, coronal, and sagittal representation of 6 µm elements from group P. **e**1–**e**3 Transverse, coronal, and sagittal representation of 4 µm elements from group Q. **f** Minimum phase projection of the entire volume reveals global structure but shows hollow centres in larger features (e.g., groups **E**–**H**), consistent with known limitations of in-line holography in recovering low-frequency phase components. **g** Sagittal cross-section through group P and Q elements. Mild reconstruction artifacts above/below structures are attributed to the limited angular coverage (missing cone). See Visualization 1 for a full 3D rendering of the phase test reconstruction

To experimentally evaluate the system’s transverse and axial resolution in a large volume, we reconstructed a custom phase resolution target with etched features down to 2 µm width, Fig. 2. The target was inclined (~25°) to imitate a 3D object and enable axial sectioning verification. Phase reconstructions revealed clear resolution of 3 µm-wide elements, while 2 µm elements remained unresolved, consistent with the 2.4 µm sensor pixel pitch. Slight artifacts observed in axial cross-sections are attributed to the limited illumination angle range. Figure 2f displays the minimum phase projection of the 3D volume along the axial direction, demonstrating the full-field overview of high-fidelity imaging.

**Fig. 3:**
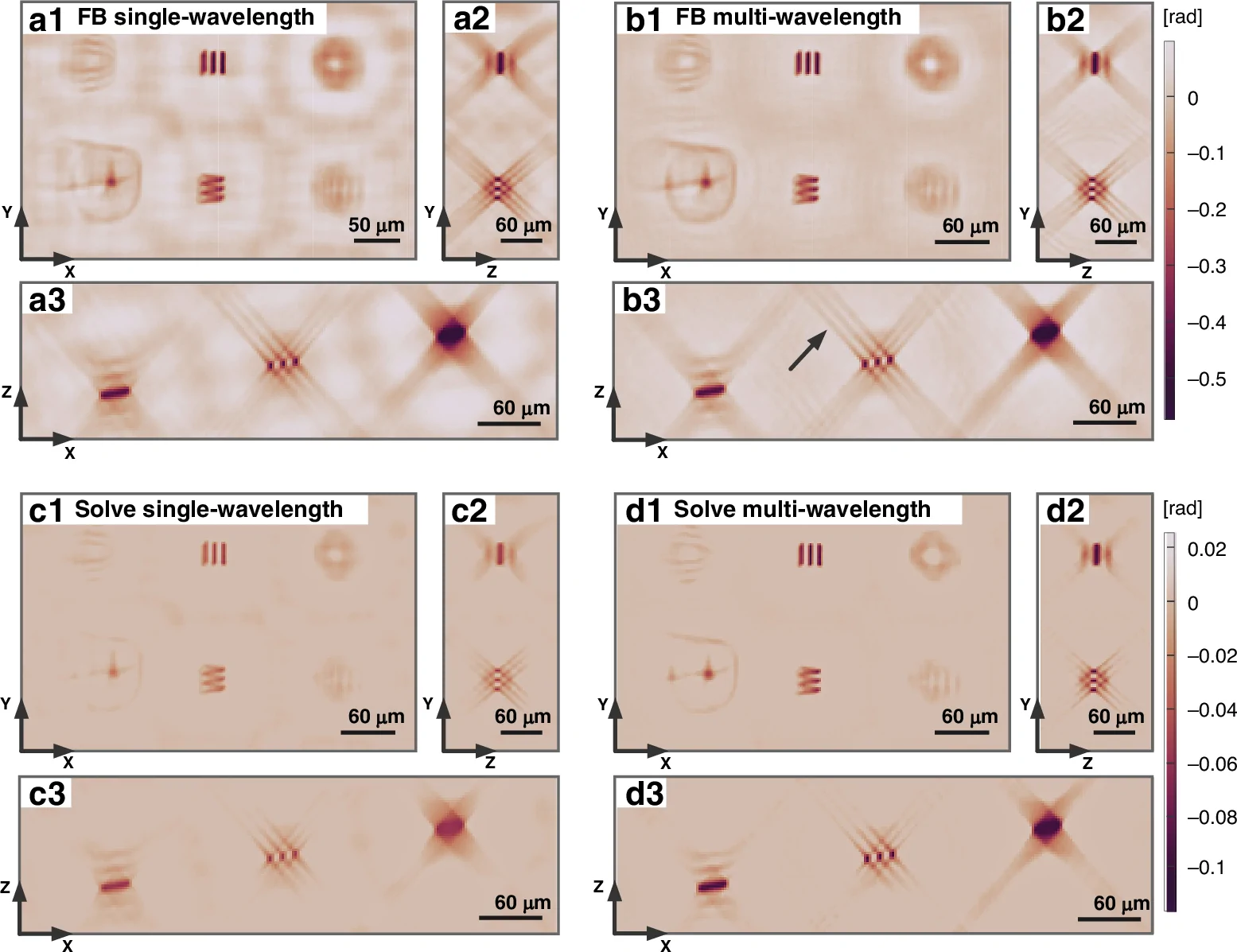
Impact of the proposed SOLVE algorithm and multi-wavelength reconstruction on 3D imaging accuracy. Selected XY (1), YZ (2) and XZ (3) slices through 3D phase reconstructions based on: **a** intensity-only holograms and **b** GS retrieved complex fields under single-scattering model (FB method). **c** intensity-only holograms and **d** GS retrieved complex fields under multiple scattering model (SOLVE method). The use of complex-field modelling and multiple scattering propagation notably improves image fidelity, sharpness, and suppression of artifacts, particularly along the axial dimension. Further comparisons of the FB and SOLVE algorithms applied to a dense, multiple scattering object are provided in Supplementary Note 2

Figure 3 presents transverse and axial cross-sections of the P-group elements from the tilted resolution phase target shown in Fig. 2, reconstructed using the FB method (Fig. 3a, b) and the proposed SOLVE algorithm (Fig. 3c, d), for both single-wavelength (Fig. 3a, c) and multi-wavelength (Fig. 3b, d) data. In the single-wavelength case, reconstructions were obtained using the same FB and SOLVE procedures, but with intensity-only input fields $C\left(x,y\right)$ estimated as the square root of the recorded intensity holograms ${I}_{{\lambda }_{1}}$, without any GS-based phase retrieval. All methods show out-of-plane artifacts (e.g., highlighted with an arrow in Fig. 3b3), which are primarily due to the limited range of illumination angles^34–36^. However, in the reconstructions obtained with the proposed SOLVE algorithm, these artifacts are significantly suppressed confirming the improved axial accuracy capability of the SOLVE method. Moreover, the use of an additional wavelength, combined with the oblique-illumination GS-based phase retrieval, effectively reduces twin-image and background-related artifacts. This leads to improved phase background uniformity and overall tomographic reconstruction quality.

**Fig. 4:**
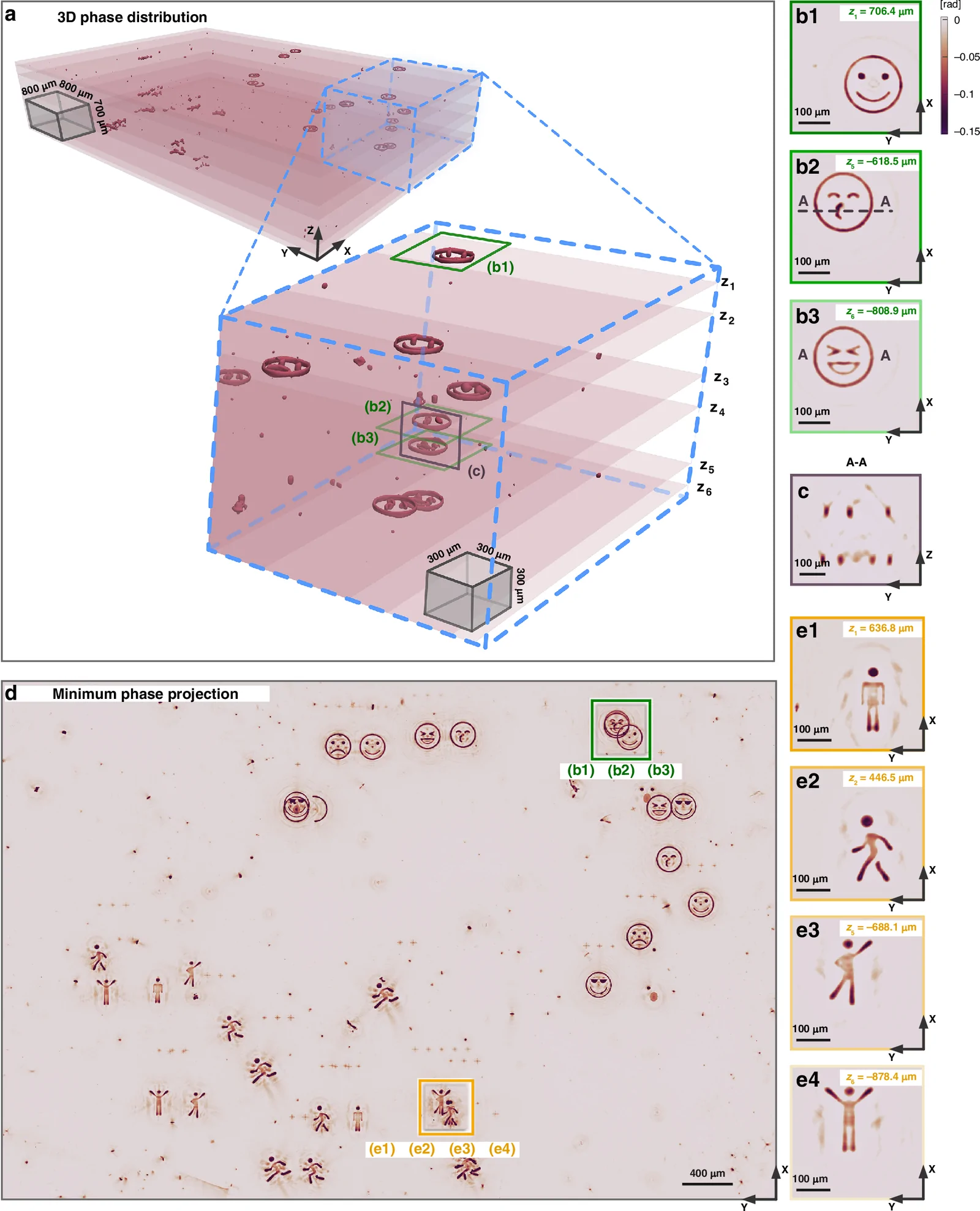
Phase reconstruction of a bespoke multilayer 3D phase test sample. **a** 3D rendering of the reconstructed phase distribution. **b**1–**b**3 Transverse phase slices showing three distinct emoji patterns located at the same lateral position, separated along the z-axis. **c** Longitudinal cross-section through two emoji patterns from **b**2 and **b**3. **d** Minimum phase projection in z direction. **e**1–**e**4 Transverse regions of four different stick figures, each located at a different axial plane. A discrepancy in the axial position of the z₁ plane can be observed between **b**1, where *z*_1_ = 706.4 μm, and **e**1, *z*_1_ = 636.8 μm. This difference arises from a slight unintentional tilt introduced to the 3D test sample during the measurement process. Visualization 2 shows a full 3D rendering of our reconstruction

Figure 4 presents the reconstruction of our bespoke 3D test sample composed of multiple phase objects arranged at different axial positions. Results demonstrate the capability of the proposed method to perform optical sectioning and distinguish multiple two-dimensional phase structures positioned at different depths, while effectively minimizing defocus contributions from out-of-focus planes. Successful 1.7 mm deep imaging of 25 mm^2^ FOV experimentally verifies gigavoxel-scale space-bandwidth product of our method.

**Fig. 5:**
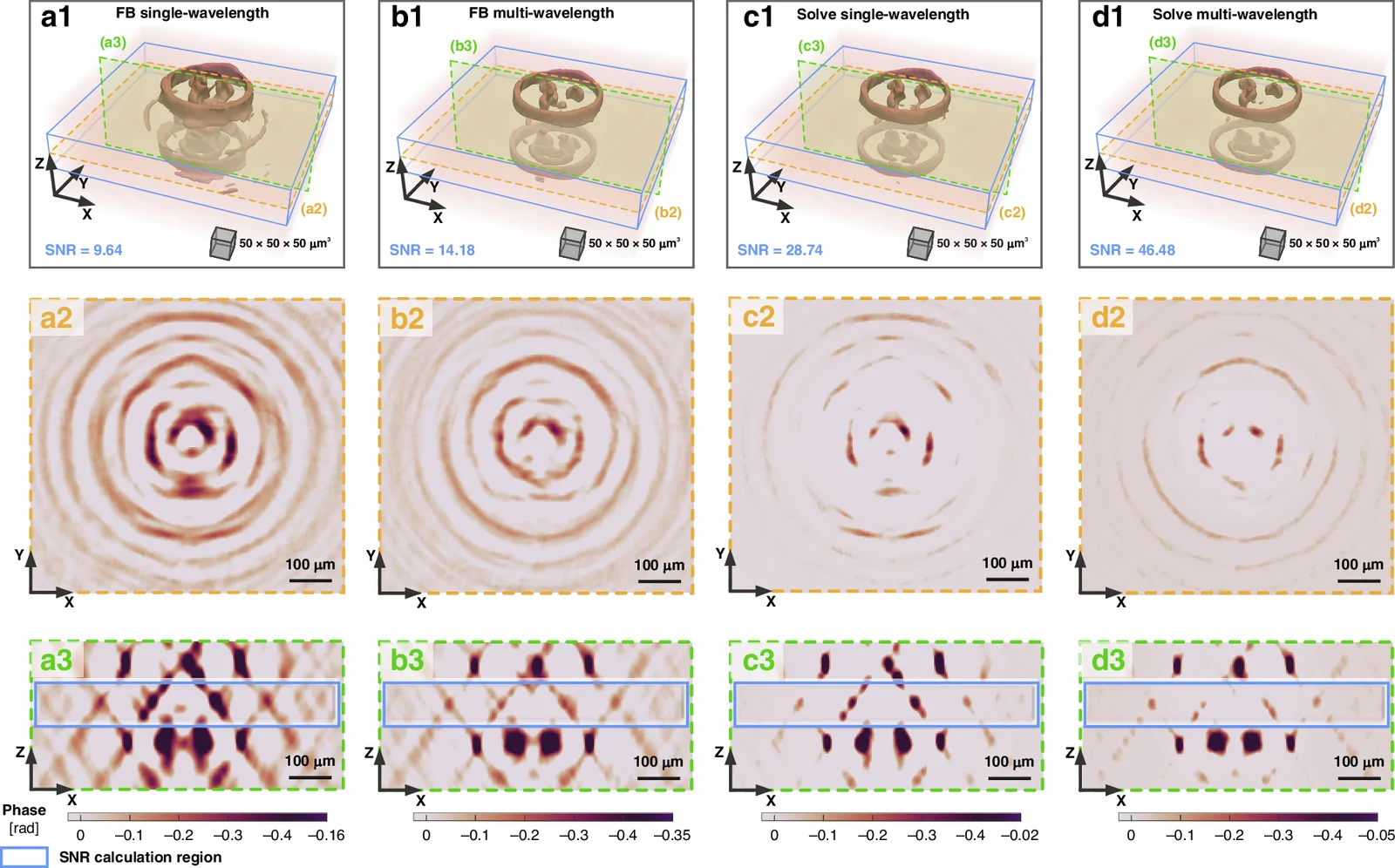
Quantitative comparison of reconstruction methods via multi-layer custom volumetric test target imaging. Results were obtained via different reconstruction schemes: **a**1 single-wavelength FB, **b**1 double-wavelength FB, **c**1 single-wavelength SOLVE, **d**1 double-wavelength SOLVE. Panels **a**2–**d**2 show respective horizontal sections marked by orange planes, and panels **a**3–**d**3 show respective vertical sections marked by green planes

Figure 5 presents quantitative comparison of holotomographic reconstruction methods via the multi-layer custom volumetric test target imaging. We targeted inter-layer object-free area (marked with blue color in Fig. 5) and calculated signal to noise ratios (SNR), as mean object phase value divided by standard deviation of the inter-layer object-free area, to quantitatively support visual examination. The higher the SNR value, the higher the quality of reconstruction as inter-layer area is object-free and should remain constant and signal-free in reconstruction. We investigated four different reconstruction schemes: single-wavelength FB in Fig. 5a1, dual-wavelength FB in Fig. 5b1, single-wavelength SOLVE in Fig. 5c1 and dual-wavelength SOLVE in Fig. 5d1. Additional panels Fig. 5a2–d2 show respective horizontal sections marked by orange planes, and Fig. 5a3–d3 show respective vertical sections marked with green planes. The object-free areas where standard deviation and SNR values were calculated are marked by blue lines. All presented panels visually align with the quantitative SNR values promoting double-wavelength SOLVE as the best reconstruction scheme due to the twin-image removal and multi-slice approach correcting for multiple scattering effect. Our observations indicate that adopting a double-wavelength strategy improves performance in both scenarios. Additionally, SOLVE achieves approximately a three-fold SNR improvement over FB.

**Fig. 6:**
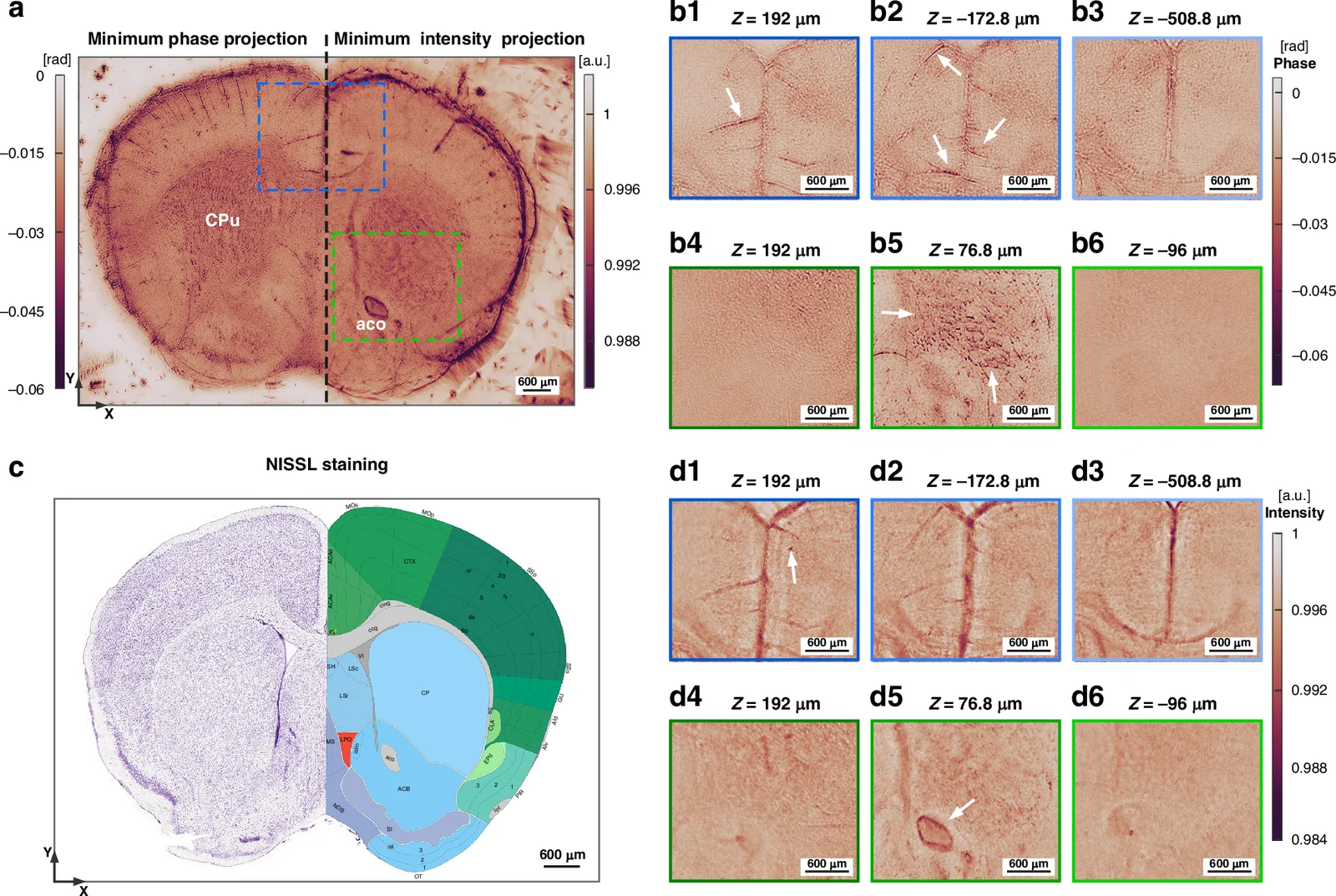
Reconstruction of a 500 μm thick optically cleared (CUBIC protocol) mouse brain section. **a** Minimum phase and intensity projections of the left and right hemisphere, respectively, in the transverse plane indicating CPu – caudoputamen and aco – anterior commissure olfactory. **b**1–**b**6 Transverse phase slices showing fine phase anatomical details (white arrows), separated along z-axis, in areas marked by dashed blue and green rectangles in **a**. **c** Corresponding Nissl-stained section (on the left) next to the brain map from the Allen Brain Atlas (on the right)^39^. **d**1–**d**6 Transverse intensity slices showing fine amplitude anatomical details (white arrows), separated along z-axis, in areas marked by dashed blue and green rectangles in **a**. Visualization 3 shows the complete tomographic sequence of input holograms

To demonstrate the capability of our system in imaging thick, morphologically-rich biological tissues, we performed a 3D reconstruction of a 500 µm-thick mouse brain section optically cleared using the CUBIC protocol^37,38^. By omitting a step when samples are cut into thin sections it is possible to crucially preserve long projections between distant brain regions. The reconstructed volume – Fig. 6 – reveals distinct morphological features depending on the imaging contrast. Blood vessels, anatomical boundaries, and small structures are highlighted differently in amplitude and phase reconstructions, emphasizing the complementary nature of both modalities. The panels in Fig. 6b1–b6 and d1–d6 present detailed minimum phase and intensity projections across localized regions and axial depths. Each panel integrates several adjacent *z*-slices centered around key structural planes to preserve continuity and detail. Vascular features are clearly visible in phase, e.g., Fig. 6b1–b2, and largely absent in amplitude, Fig. 6d1–d2. Figure 6d1 shows an imaging artifact unique to amplitude modality, not observed in the corresponding phase projection. Notably, the anterior commissure olfactory (aco) is well defined in the amplitude image at a specific axial plane Fig. 6(d5), while the caudoputamen (CPu) appears distinctly only in phase (b5), at Z = 76.7 µm, and is not visible at neighboring depths, Fig. 6(b4) and (b6), underscoring the high depth resolution of our approach. For anatomical reference, panel Fig. 6(c) includes a Nissl-stained histological section and corresponding annotated map from the Allen Mouse Brain Atlas^39^.

The mouse brain section had in-plane dimensions of 9.5 mm × 7 mm, slightly exceeding the FOV of our system in the *y*-direction. As a result, portions of the diffracted light fell outside the camera sensor area (see Visualization 3 for full holographic sequence); however, each region of the sample was covered in at least three-quarters of collected projections. This redundancy enabled 3D reconstruction of the entire section without the need for mechanical stitching or sample/camera motion. The sample was immersed in CUBIC-R1 solution during measurement to reduce refractive index mismatch.

In lensless holotomography, defocused acquisition combined with tilted illumination produces a characteristic transverse projection shift - an effect that can enlarge the effective FOV (one side enlargement up to $2{z}_{{co}}\cdot \tan (\alpha )$; in brain imaging case it is 3.1 mm for *z*_*co*_ *=* 1.72 mm and *α* *=* 42^∘^) but may also truncate portions of the object in some azimuth ranges. This shift/truncation mechanism is generally absent in lens-based tomographic imaging, where projections are captured in the image plane, remaining in focus and centered. A 3D transfer-function analysis confirms that such truncation has limited impact on recoverable information, see Supplementary Note 7; Fig. S7, with only ~3% loss in transferred spectrum volume for the mouse-brain-like condition and ~17% spectral loss for a simulated 50% missing-projection stress test.

## Discussion

The LHT systems are generally limited in resolution by the pixel size, however demonstrate overall superior tomographic imaging capability, as their transfer functions – analyzed in detail in Supplementary Note 1 – are much less anisotropic than for the lens-based HT. The LHT, with approx. 40 degrees cone illumination, thus provides better, i.e., more isotropic, coverage of the 3D object spectrum suggesting quasi-isotropic hardware-limited (pixel size – 2.4 µm in our case) resolution in XYZ (transversal and axial). The FOV in LHT is, in principle, limited only by the physical dimensions of the camera sensor (13.2 mm × 8.8 mm in our case). Diffraction patterns originating near the edges of the sample may, however, fall outside the camera sensor area for certain illumination directions due to defocused imaging geometry. Despite this fundamental information reduction, the achievable effective FOV remains significantly larger than that of conventional lens-based HT systems (up to around 7000 times larger comparing to commercial Nanolive system).

Standard multiple scattering tomographic reconstruction algorithms^31,40–43^ iteratively improve the 3D reconstruction by minimizing the discrepancy between the experimentally acquired data and the model predictions using gradient descent minimization and are generally computationally expensive. Proposed SOLVE approach improves the 3D reconstruction by iteratively backpropagating the optical field corrections, given by a difference between the multi-slice model prediction of the optical field and the actual acquired data. A conceptually strategy has previously been employed only in lens-based systems^44–47^. This alternative error-reduction mechanism for multiple scattering tomography has been demonstrated to be effective and computationally efficient^48^ laying foundation for our SOLVE method. SOLVE is implemented in space domain thus it eliminates the need for direct filling of the 3D Fourier space and – crucially for large-volume imaging – avoids the related interpolation errors. Additionally, space-domain implementation enables tackling the problem of deterioration of the reconstruction accuracy in the peripherical regions of the tomographic volume that is typical for Rytov-based direct inversion algorithms^32,49^. Moreover, it offers the potential to accommodate arbitrarily shaped illumination beams – particularly spherical beams, useful in compact LHT. As diffraction tomographic reconstruction is generally a computationally demanding, in SOLVE we have specialized algorithm implementation and optimization routines in place, described in detail in Supplementary Note 8, aiming at efficient large volume computations.

Our LHT SOLVE framework reconstructs a complex-valued scattering potential, and therefore naturally supports samples exhibiting both refractive-index (phase) and absorption (amplitude) contrast within the same reconstruction. This is reflected in the experiments shown here, which span distinct regimes: (i) weakly absorbing, nearly index-matched two-photon-polymerized structures embedded in immersion oil, behaving predominantly as phase multi-layer objects while still inducing multiple scattering; and (ii) a 500-µm-thick CUBIC-cleared mouse brain slice, where residual refractive-index mismatch and absorption lead to pronounced multiple scattering, and where we report both phase and amplitude reconstructions (Fig. 6). Practically, the background refractive index ${n}_{i}$ is a user-defined parameter in the forward model, thus adapting SOLVE to different immersion media requires no hardware changes - only an updated value of ${n}_{i}$. The primary regime-of-validity constraint is the in-line holographic (Gabor) recording condition: if absorption and/or scattering become too strong, the measured intensity no longer supports reliable phase retrieval under the assumed model, independent of sample thickness per se. Finally, the SHARP calibration explicitly compensates Snell-law induced lateral shifts caused by refractive-index mismatch between air, the sample, and the cover glass, facilitating robust operation across different mounting and clearing configurations.

A known limitation of in-line (Gabor) holographic phase retrieval is the reduced transfer of low spatial frequencies in the recovered phase. Because the measured hologram intensity is dominated by the unscattered reference wave, slowly varying phase terms contribute only weakly to the recorded contrast and can therefore be partially suppressed or ambiguously recovered, particularly in single-shot reconstructions. Additionally, contrast transfer function approaches zero for very low spatial frequencies. This limitation persists even when using iterative two-hologram Gerchberg–Saxton type schemes: while iterative constraints improve stability and reduce twin-image artifacts, the recovery of very low-frequency phase components can remain incomplete, which can compromise strictly absolute, fully quantitative phase readout. Importantly, however, mid-to-high spatial frequencies – which carry the majority of structural details – typically generate high contrast hologram features upon defocusing and thus are transferred with high fidelity, so the approach remains well-suited for resolving fine features and for comparative/relative phase imaging across the volume, with the note on Gabor holography related fundamental low frequency problem.

A key advantage of the lensless arrangement is the large field of view, but in the present implementation the lateral resolution is sampling-limited by the detector pixel pitch (Δ_*x*_ *=* 2.4 um). In lens-based optical diffraction tomography and related tilted-illumination schemes, varying the illumination angle can increase lateral resolution via a synthetic-aperture mechanism that expands the accessible spatial-frequency support. In our current LHT geometry, however, the effective bandlimit is set primarily by the camera sampling: once the measurement is pixel-limited, tilting the illumination does not recover higher transverse spatial frequencies beyond those that can be faithfully sampled on the sensor, and thus does not lead to a net resolution gain. Resolution enhancement would become feasible in regimes where sampling no longer dominates: for example by using a detector with a sufficiently smaller pixel pitch, introducing an appropriate magnification stage (at the expense of FOV), or employing pixel super-resolution strategies that fuse multiple sub-pixel-shifted holograms to reduce the effective pixel size ${\Delta }_{x}$. Exploring such sampling-relief approaches to reach a more conventional $\lambda /{{\rm{NA}}}$-limited regime is an interesting direction for future work.

The supercontinuum source provides tunable illumination with a wavelength-dependent spectral bandwidth; over 400–650 nm the specified full-width half maximum spans ~1.8–8.5 nm. Although shorter visible wavelengths can improve diffraction-limited lateral resolution, the present system is predominantly limited by detector sampling (pixel size) rather than diffraction, so shifting to violet would not yield a meaningful resolution gain under the current conditions. The choice of 540 nm and 640 nm therefore reflects a practical trade-off that also benefits from higher available optical power at longer wavelengths, improving the signal-to-noise ratio of the recorded holograms. Once the sampling limit is mitigated (e.g., by pixel super-resolution or a smaller-pitch sensor), shorter wavelengths are expected to translate into measurable resolution improvement.

By addressing fundamental optical and computational challenges, our LHT approach enables high-fidelity, large-volume 3D imaging with multiple scattering correction, previously unattainable without complex optics and laborious scanning approaches. The integration of scalable hardware with the new reconstruction and calibration algorithms establishes a versatile platform suitable for diverse applications across biology, medicine, and materials science. We anticipate that this framework may inspire further innovations in lens-free volumetric microscopy and expand accessibility to high-throughput label-free advanced 3D imaging.

## Materials and Methods

### Experimental setup

Proposed experimental setup consists of a board-level CMOS camera (ALVIUM 1800 U-2050 m mono Bareboard, 2.4 μm pixel size, 5496 × 3672 resolution), the measured sample (positioned around 2 mm above the camera sensor), a collimating lens CL (*f* = 200 mm) and a coherent illumination light source LS (Supercontinuum laser, NKT Photonics SuperK EVO), delivered to the system with a single-mode optical fiber (Thorlabs P1-S405-FC-2, core diameter: ~3 μm), see Fig. 1a. The output of the fiber is mounted on a motorized rotation stage (Standa 005460), enabling precise angular scanning of the illumination beam across a full 360° range. Illumination angle is easily adjustable and was generally set between 35° and 45° to ensure quasi-isotropic XYZ reconstruction, while maintaining relatively large FOV. During the dataset acquisition, the rotation stage steps in 2° increments resulting in *N* = 180 angular positions. At each position, two holograms are recorded – one using ${\lambda }_{1}=540$ nm and the other ${\lambda }_{2}=640$ nm illumination wavelength – resulting in a total of 360 images per dataset. Additionally, one on-axis hologram (with 540 nm wavelength) is registered only for angle calibration purposes. The data acquisition step was not hardware nor software optimized and it lasted around 30 minutes in current proof-of-concept version of the setup.

### 2PP volumetric test target fabrication

Two-photon polymerization target consists of three high-precision coverslips (each 170 µm thick, with *n*_e_ = 1.5255 ± 0.0015), resulting in six discrete test planes (z1–z6 in Fig. 2). Each plane contains distinct patterns of 30 µm thick emojis and stick figures, fabricated using a two-photon polymerization technique (Photonic Professional GT2, Nanoscribe GmbH) on both sides of coverslip (0.17 mm thick). The structures were designed in the DeScribe software and composed of IP-S photoresist (refractive index around 1.515 when fully polymerized), selected for its nearly pure phase characteristics due to its polymeric nature. The coverslips are spaced using 430 µm thick spacers and immersed in *n*_e_ = 1.518 Nikon Type N immersion oil. Additional clean coverslips are placed on the top and bottom of the sample bringing the total thickness of the 3D sample to approximately 1.7 mm (largest distance between two printed structures is 1.37 mm).

### Mouse brain sample preparation

Brain was collected from the adult C57Bl6 mouse. Mouse was anesthetized using overdose of sodium pentobarbital. Next it was perfused as described previously^50^. Brain tissue was collected, postfixed overnight in 4 °C. Next, it was embedded in 4% agarose to facilitate cutting. Brain was cut using vibratome (Leica) into 500 µm sections and stored for the analysis in PBS solution in 4 °C. Tissue optical clearing was performed using the CUBIC-R1 solution, prepared according to a published protocol^37^. A clearing solution was prepared as a mixture of 25% (mass fraction) urea (Sigma-Aldrich), 25% (mass fraction) N; N; N0; N0-tetrakis(2-hydroxypropyl)ethylenediamine (Sigma-Aldrich), and 15% (mass fraction) Triton X-100 (Sigma-Aldrich) dissolved in distilled water. For tissue optical clearing, the agarose was removed from the brain slices, which were then submerged in an excess (around 5 ml) of CUBIC-R1 solution in a custom-made Petri dish to minimize the potential effects of solution evaporation.

## Supplementary information

Supplementary Document 1

Full 3D rendering of the phase test reconstruction (Fig. 2)

Full 3D rendering of a bespoke multilayer volumetric phase test sample (Fig. 3)

Complete tomographic sequence of input holograms for brain tissue slice imaging (Fig. 6)

## Data Availability

The raw data supporting the findings of this study, including all hologram series used in Figs. 2, S2, 3, 4, 5, are available at Zenodo: https://doi.org/10.5281/zenodo.18771332.

## Code availability

The algorithms used in this study are provided as part of the Supplementary Information.
