# Supplementary material for: Gigavoxel-scale multiple-scattering-aware lensless holotomography
Source: Light Sci Appl. 2026 Sep 16;15:381. doi: 10.1038/s41377-026-02416-0 (PMC13578224; doi:10.1038/s41377-026-02416-0)
Supplement: Supplementary file 1 [file 41377_2026_2416_MOESM1_ESM.pdf]

Supplementary Document 1

A supplementary document for:

Gigavoxel-Scale Multiple-Scattering-Aware Lensless Holotomography

Mikołaj Rogalski[1,~], Julianna Winnik[1,~], Julia Dudek[1], Piotr Arcab[1], Emilia Wdowiak[1], Paweł Matryba[2,3], Marzena Stefaniuk[2], Piotr Zdańkowski[1], Maciej Trusiak[1,*]

[1]Warsaw University of Technology, Institute of Micromechanics and Photonics, Faculty of Mechatronics, Warsaw, Poland

[2]Laboratory of Neurobiology, Nencki Institute of Experimental Biology of Polish Academy of Sciences, Warsaw, Poland

[3]Department of Immunology, Medical University of Warsaw, Warsaw, Poland

~co-first authors

*maciej.trusiak@pw.edu.pl

Supplementary note 1: Theoretical transfer functions, resolution and field of view analysis

This part analyses the tomographic imaging capability of conventional lens-based holotomography (HT) and lensless holotomography (LHT) by comparing their 3D transfer functions (TF). The TFs presented in Fig. S1 were obtained using identical system parameters: a conical illumination scenario with an incidence angle of $\alpha = 40°$, a light wavelength of $\lambda = 0.54$ µm, and a refractive index of the surrounding medium of $n_i = 1$.

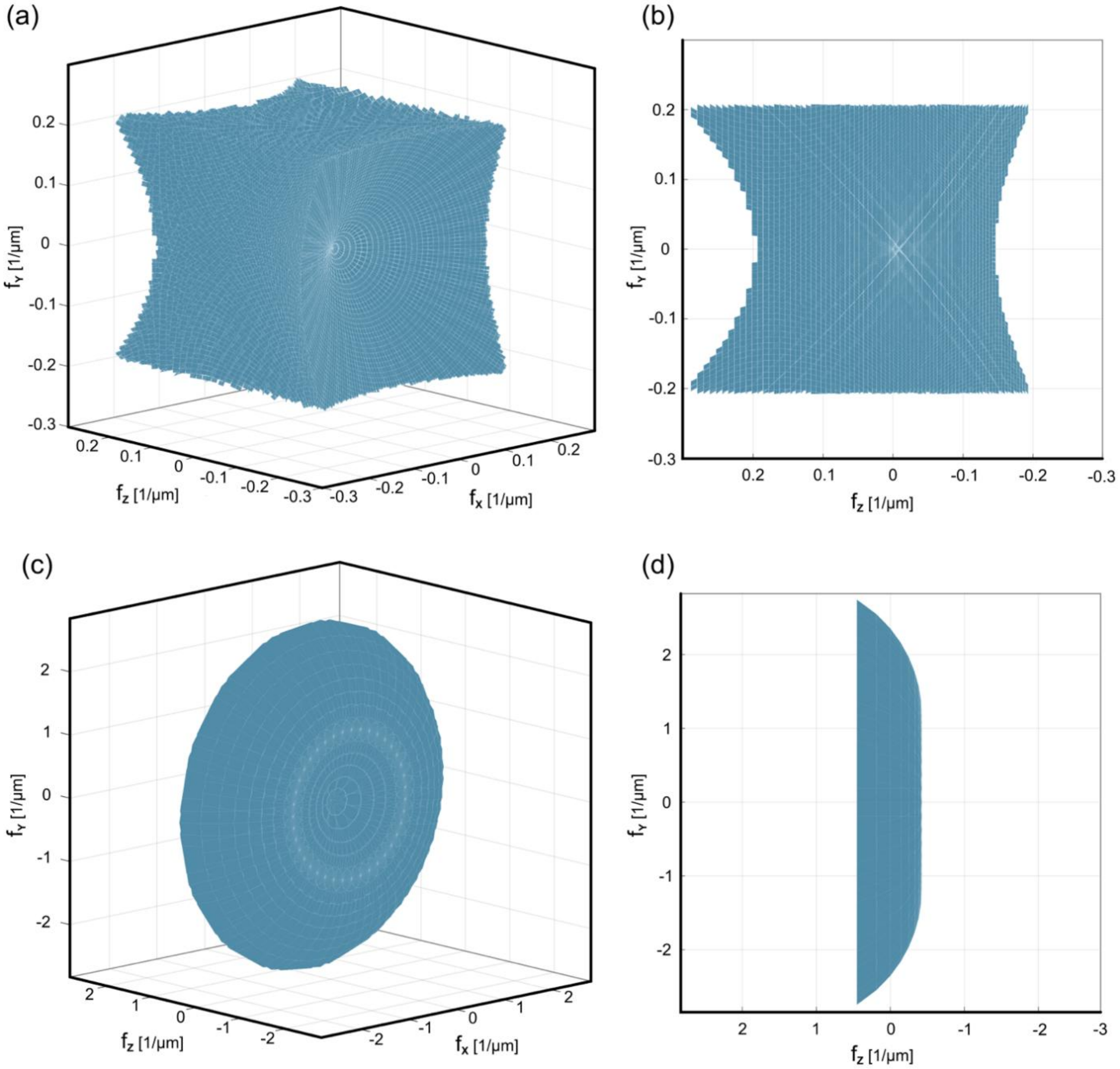

Fig. S1. Two 3D renderings of transfer functions for tomographic imaging with lensless (a, b) and conventional lens-based systems (c, d). The applied parameters are as follows: a conical illumination scenario with an incidence angle of $40°$, light wavelength of $\lambda = 0.54$ µm, and a refractive index of the surrounding medium of $n_i = 1$. The transverse resolution is limited by the pixel size (here $\Delta_x = 2.4$ µm) in the lensless system (a) and by the numerical aperture (here $NA = 0.85$) in the lens-based setup (c). Lensless tomographic imaging provides a favorable quasi-isotropic imaging condition, as evidenced by comparison of the transfer functions extents in (b) and (d).

In the typical lensless tomography system, the transverse resolution is limited by the pixel size Δ_x; therefore, the spatial frequency limit of 3D imaging in the transverse direction is given by ${f_x}^{LHT} = 1/(2\Delta_x)$ (here $\Delta_x = 2.4$ µm thus ${f_x}^{LHT} = 0.208$ µm^{-1}). Notably, in lensless systems, this frequency limit is independent of the illumination angle. This also means that steep illumination angles, which are required for tomographic reconstruction, can be applied even in relatively low-resolution systems. In contrast, for lens-based systems, the maximum transferred spatial frequency ${f_x}^{HT}$ depends on both the numerical aperture (NA) of the imaging system and the applied illumination angle: ${f_x}^{HT} = (NA + n_i sin\alpha)/\lambda$. Moreover, in lens-based system the illumination angle must fall within the NA-related acceptance angle: $\alpha < \alpha_{NA} = \operatorname{asin}(NA/n_i)$, which imposes the requirement for high-NA optics.

For our comparative analysis (Fig. S1), we assumed an illumination angle of $40°$, which matches the value used in our experimental setup. To apply the same angle in a lens-based tomographic system, the numerical aperture must satisfy $NA > sin(40°) \approx 0.643$. In our simulation, we assumed $NA = 0.85$, which corresponds to an optics used in a widely used, commercial lens-based HT system by Nanolive. Under these conditions ${f_x}^{HT} = 2.76$ µm^{-1}. It is important to note that high NA must be accompanied by large transverse magnification (60x for Nanolive), which results in a small FOV (85 x 85 µm for Nanolive). In contrast, the lensless system supports steep illumination while maintaining small (unity) magnification enabling tomographic imaging over significantly larger FOV (sensor size dependent, however easily up to 5 x 5 mm), allowing for whole-slide tissue slice and cell culture imaging without the time-consuming and quality-decreasing need for scanning and stitching.

Lastly, the LHT system demonstrates overall superior tomographic imaging capability, as its TF is much less anisotropic than for the lens-based HT, see Fig. S1. Notably, both considered systems (lensless and lens-based) utilize the same limited-angle tomographic architecture, which results in analogical missing cone problem, i.e., the conical region centered around the f_z axis where object information is missing. However, as seen from comparison of the axial width of LHT and HT in Figs. S1(b) and S1(d), the lensless imaging system provides better, i.e., more isotropic, coverage of the 3D object spectrum. In more detail, the extension of lensless tomography TF for positive and negative axial frequencies is given by the following limits:

$$
{f_z}^{LHT} = \begin{cases} \frac{n_i}{\lambda} cos\alpha - \sqrt{\left(\frac{n_i}{\lambda}\right)^2 - \left(\frac{\sqrt{2}}{2\Delta_x} + \frac{n_i}{\lambda} sin\alpha\right)^2} & \text{for positive } f_z \\ \frac{n_i}{\lambda} cos\alpha - \sqrt{\left(\frac{n_i}{\lambda}\right)^2 - \left(\frac{\sqrt{2}}{2\Delta_x} - \frac{n_i}{\lambda} sin\alpha\right)^2} & \text{for negative } f_z \end{cases} . \tag{S1}
$$

The corresponding half-width is given by:

$$
{HW_z}^{LHT} = 0.5\left[\sqrt{\left(\frac{n_i}{\lambda}\right)^2 - \left(\frac{\sqrt{2}}{2\Delta_x} - \frac{n_i}{\lambda} sin\alpha\right)^2} - \sqrt{\left(\frac{n_i}{\lambda}\right)^2 - \left(\frac{\sqrt{2}}{2\Delta_x} + \frac{n_i}{\lambda} sin\alpha\right)^2}\right], \tag{S2}
$$

which for the given parameters results in ${HW_z}^{LHT} = 0.257$ µm^{-1}. Notably, in this case, the axial extent of 3D transfer function is even slightly larger than the traverse extent (${f_x}^{LHT} = 0.208$ µm^{-1}).

For comparison, the axial half-width of lens-based HT transfer function (assuming conical illumination scenario) is given by:

$$ {HW_z}^{HT} = \frac{n_i}{\lambda}\left(sin\frac{\alpha_{NA}}{2}\right)^2, \quad (S3) $$

which for the considered parameters gives ${HW_z}^{HT} = 0.438$ µm^{-1}. This means that the transverse extent of TF is approximately 6 times larger than its axial dimension: ${f_x}^{HT} = (NA + n_i sin\alpha)/\lambda = 2.76$ µm^{-1}.

In summary, we showed that the extended axial imaging depth of LHT, which is demonstrated in main article, is accompanied by relatively strong depth discrimination (tomogram axial resolution), enabled by the unique off-axis defocused imaging mechanism of lensless holographic microscopy.

Supplementary note 2: Comparison of LHT reconstruction algorithms for multiple scattering objects

Figure S2 presents a comparison of the 3D reconstruction of a Colosseum model structure using a two-photon polymerization system. The STL model[S1] was translated into printing coordinates and fabricated using the IP-S photoresist (Nanoscribe). The structure measures 500 × 400 × 134 µm^3 (length × width × height) and is characterized by a refractive index of 1.515. It was fabricated on a high-precision 170 µm-thick coverslip and immersed in Nikon Type N immersion oil (refractive index n_e = 1.518). Figures S2(a)–S2(d) show 3D visualizations of reconstructions obtained using the filtered backpropagation (FB) method (Figs. S2(a), S2(b)) and the proposed SOLVE method (Figs. S2(c), S2(d)), for both single-wavelength (Figs. S2(a), S2(c)) and multi-wavelength (Figs. S2(b), S2(d)) datasets. The visualizations were generated using Tomviz software, with threshold cutoff values set to 0.1165 rad (Fig. S2(a)), 0.1663 rad (Fig. S2(b)), 0.0146 rad (Fig. S2(c)), 0.0228 rad (Fig. S2(d)) – all thresholds were set manually to highlight the structure of the object while minimizing visible missing-cone related artifacts. Figure S2(e) shows the ground truth STL model used for two-photon polymerization printing of the test structure.

As shown, reconstructions based on single-wavelength data suffer from prominent twin-image artifacts, particularly visible in the empty central region of the Colosseum structure (highlighted with blue ellipse in Figs. S2(a) and S2(c)). In contrast, the use of multi-wavelength data significantly suppresses these artifacts, enabling more accurate separation of object information from the background during thresholding.

Further comparison between the FB and SOLVE reconstructions reveals marked differences in reconstruction fidelity. The FB-based reconstruction displays structural heterogeneity: certain

regions appear substantially thicker than their SOLVE counterparts (green regions in Figs. S2(b) and S2(d)), while other parts present in the SOLVE result are completely missing from the FB reconstruction (yellow regions in Figs. S2(b) and S2(d)). These discrepancies highlight the higher fidelity of the SOLVE reconstruction, as its greater uniformity is more consistent with the fact that the printed object had a homogeneous refractive index.

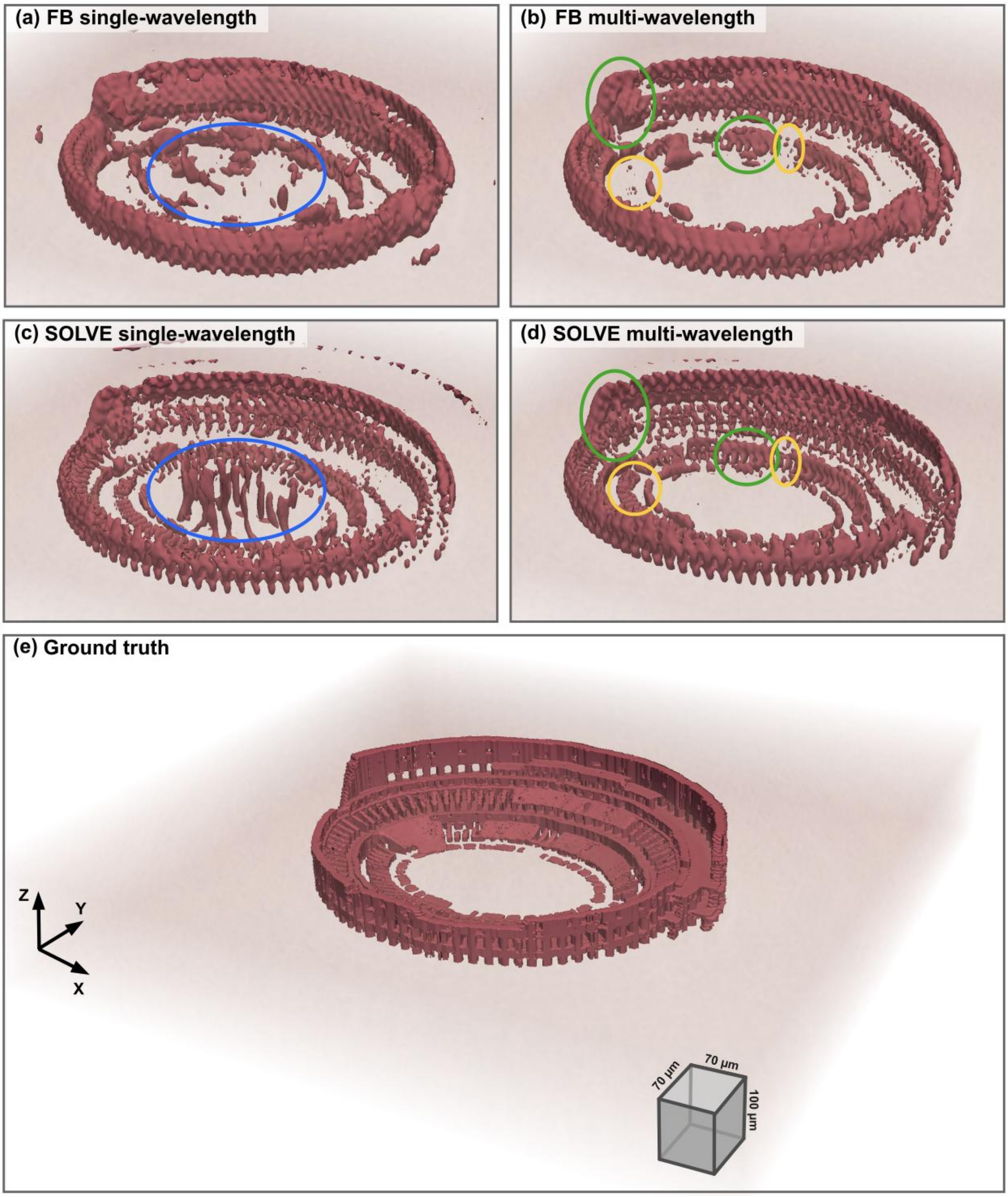

Fig. S2. 3D phase reconstructions of Colosseum model fabricated with two-photon polymerization visualizing the impact of the proposed SOLVE algorithm and multi-wavelength reconstruction on 3D imaging accuracy. Reconstructions based on: (a) intensity-only holograms and (b) GS retrieved complex fields under single-scattering model (FB method). (c) Intensity-only holograms and (d) GS retrieved complex fields under multiple-scattering model (SOLVE method). (e) Ground truth STL model[S1].

Supplementary note 3: Tailored angular spectrum propagation algorithm for lensless holotomography

A critical aspect of holographic reconstruction in lensless microscopy involves backpropagating the optical field captured at the camera plane to the object plane. In lensless microscopy systems with non-unity magnification – where the curvature of the illumination wavefront cannot be neglected – specialized numerical refocusing algorithms with adaptive sampling intervals are employed[S2-S4]. In our system, however, collimating lens is used resulting in unity magnification.

Nonetheless, a significant challenge arises due to the steep illumination angles required by the tomographic reconstruction algorithm. Our reconstruction algorithm is based on a widely adopted method utilizing optical field decomposition into plane waves, known as the Angular Spectrum (AS)[S5]. The first step of the AS method is to compute the two-dimensional Fourier transform of the optical field $U_{z=0}$ in the source plane $z = 0$:

$$\widetilde{U}_{z=0}(f_x, f_y) = \iint U_{z=0}(x, y)\exp[-i2\pi(f_x x + f_y y)]dxdy, \tag{S4}$$

which corresponds to the decomposition of the signal into plane waves. In the above expression, f_x and f_y represent the spatial frequencies of the object beam. Next, the product of the optical field spectrum and the free-space propagation transfer function is calculated:

$$\widetilde{U}_z(f_x, f_y) = \widetilde{U}_{z=0}(f_x, f_y)\exp\left[i2\pi z\sqrt{\left(\frac{n_i}{\lambda}\right)^2 - f_x^2 - f_y^2}\right], \tag{S5}$$

where λ is a light wavelength, n_i denotes refractive index of surrounding medium, and z is the propagation distance. The last step is inverse Fourier transform:

$$U_z(x, y) = \iint \widetilde{U}_z(f_x, f_y)\exp[i2\pi(f_x x + f_y y)]df_x df_y. \tag{S6}$$

Figure S3 illustrates the AS algorithm with simulation-based example. Since our focus is on propagation method for limited angle tomography, we consider a tilted propagation direction (α_x, α_y) that corresponds to Fourier frequencies ($f_{ix} = n_i \sin(\alpha_x)/\lambda$, $f_{iy} = n_i \sin(\alpha_y)/\lambda$) (in our example, for simplicity, we assumed $\alpha_y = 0$). The input optical field is modelled as the product of the illumination field, here, tilted plane wave $\exp[i2\pi(f_x x + f_y y)]$ (Fig. S3(e)), and the sample contribution, which in this case is centrally located circular amplitude modulation (Fig. S3(a)). We assume following parameters: light wavelength $\lambda = 0.54$ µm, propagating in a medium with refractive index $n_i = 1$ at angle $\alpha_x = 40°$ on a distance of $z = 30$ µm. The optical field is discretised with 1024 x 1024 grid and sampling interval is equal to 0.3λ but later in this section we will also consider sampling with larger interval, i.e., $\Delta = 2.4$ µm. The choice of the parameters value is motivated by the condition of the experimental system of our lensless tomogram.

The amplitude and phase of the source optical field $U_{z=0}(x, y)$ is displayed in Fig. S3(a) and S3(e), respectively. The off-axis character of $U_{z=0}$ can be seen from its phase tilt in Fig. S3(e). Figures S3(b) displays the amplitude of the spectrum $\widetilde{U}_{z=0}$ of the source field. During the AS algorithm, $\widetilde{U}_{z=0}$ is multiplied with the propagation transfer function (Figs. S3(c) and S3(f)), and

then inverse Fourier transformed to produce the output field $U_z(x, y)$ (Figs. S3(d) and S3(g)). It can be seen that the tilted propagation direction introduces numerical errors in the form of diffraction artifacts at the boundary of the computational matrix (artificial vertical lines in Fig. S3(d)). These errors result from the use of the fast Fourier transform, which assumes periodic boundary conditions and leads to discontinuities at the image edges due to the phase ramp associated with the off-axis propagation direction.

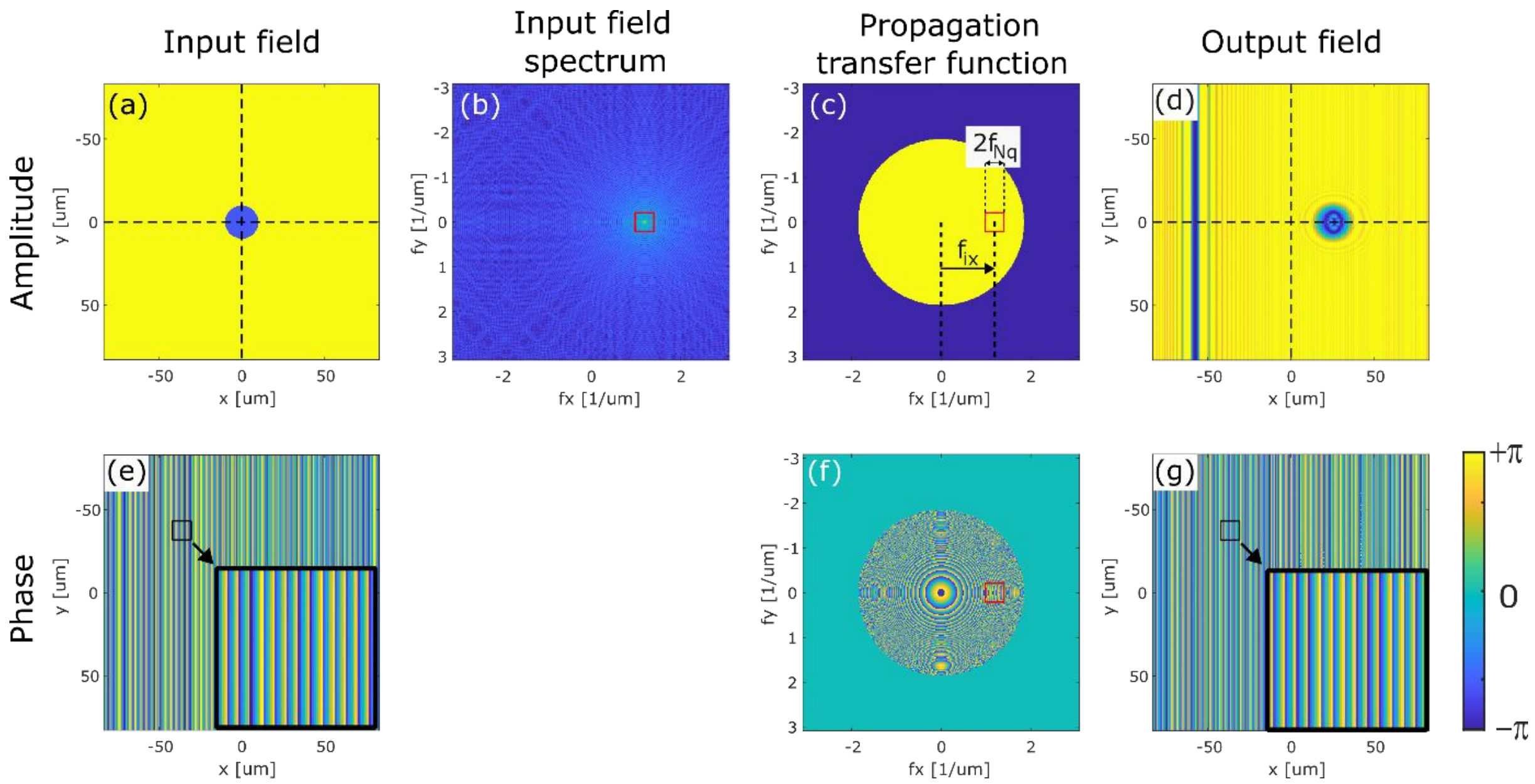

Fig. S3. Visualization of the AS algorithm: amplitude (top row) and phase (bottom row) of (a, e) the input optical field, (b) the spectrum of the input optical field, (c, f) the propagation transfer function, and (d, g) the output optical field. Results obtained for: wavelength $\lambda = 0.54$ μm, illumination angle $\alpha_x = 40°$, propagation distance $z = 30$ μm. The red rectangle indicates the Fourier frequency range that is transferred by lensless microscope with a pixel size of 2.4 μm.

The red rectangle in Fourier spectrum (Figs. S3 (b),(c),(f)) denotes the Fourier frequency range of a single object wave that is accessible with lensless microscopy, where the limiting factor for resolution is typically the camera's pixel size Δ (here we assumed $\Delta = 2.4$ μm). The resolution limitation mechanism is as follows – the pixel size must be small enough to allow the recording of interference fringes formed between the wave scattered by the object and the undisturbed illuminating wave. This implies that, unlike in classical microscopy, the frequency limitation applies to spatial frequencies shifted by the illumination frequency vector (f_{ix}, f_{iy}). In other words, the system transmits spatial frequencies within a rectangular aperture (red rectangle in Fig. S3(c)) localized around the spatial frequency (f_{ix}, f_{iy}):

$$\begin{cases} |f_x'| = |f_x - f_{ix}| < f_{Nq} \\ |f_y'| = |f_y - f_{iy}| < f_{Nq} \end{cases}, \qquad \text{(S7)}$$

where Nyquist frequency $f_{Nq} = 1/(2\Delta)$. Notably, this is a unique feature of the lensless microscopy. In the lens-based system, resolution is typically limited by the numerical aperture of the optical setup and is always cantered around the zero Fourier frequency of the object wave.

In summary, AS algorithm**,** when applied to lensless holotomography, faces two main challenges. First, inclined illumination introduces boundary artifacts. Second, the relevant Fourier components of the object waves are shifted away from the centre of the computational matrix. As a result, the algorithm requires significantly greater computational resources – namely, smaller pixel sizes and a larger number of pixels – to accurately capture the shifted spectrum.

As it will be shown, both of these issues can be effectively resolved by performing computations in shifted spatial frequencies coordinates, defined as $(f_x', f_y') = (f_x - f_{ix}, f_y - f_{iy})$. The modified algorithm is illustrated with Figure S4. First, the input object wave $U_{z=0}(x, y)$ is multiplied by a conjugate illumination plane wave, resulting in a modified source field $U_{z=0}^{lf}(x, y)$ with the phase tilt removed (Fig. S4(e)). This operation effectively shifts the object wave's spectrum to the centre of the computational matrix (superscript lf stands for low-frequency shifted wave):

$$U_{z=0}^{lf}(x, y) = U_{z=0}(x, y)\exp\left[-i2\pi\left(f_{ix}x + f_{iy}y\right)\right]. \tag{S8}$$

Next, the standard AS steps - Fourier transformation, multiplication with transfer function, and inverse Fourier transform – are carried out in the shifted spatial frequency coordinates (Fig. S4 (b),(c),(f)):

$$\widetilde{U}_{z=0}\left(f_x', f_y'\right) = \iint U_{z=0}^{lf}(x, y)\exp\left[-i2\pi\left(f_x'x + f_y'y\right)\right]dxdy, \tag{S9}$$

$$\widetilde{U}_z\left(f_x', f_y'\right) = \widetilde{U}_{z=0}\left(f_x', f_y'\right)\exp\left[i2\pi z\sqrt{\left(\frac{n_i}{\lambda}\right)^2 - f_x'^2 - f_y'^2}\right], \tag{S10}$$

$$U_z^{lf}(x, y) = \iint U_z\left(f_x', f_y'\right)\exp\left[i2\pi\left(f_x'x + f_y'y\right)\right]df_x'df_y'. \tag{S11}$$

The final result is a propagated beam with the carrier frequency removed and boundary artifacts significantly reduced (Figs. S4(d) and S4(g)). Notably, this feature of the modified AS have been already discussed in the context of application to lens-based holotmography[S6,S7] and lensless holography[S8]. If needed, the original carrier frequency can be reintroduced by multiplying U_z^{lf} by the illumination plane wave:

$$U_z(x, y) = U_z^{lf}(x, y)\exp\left[i2\pi\left(f_{ix}x + f_{iy}y\right)\right]. \tag{S12}$$

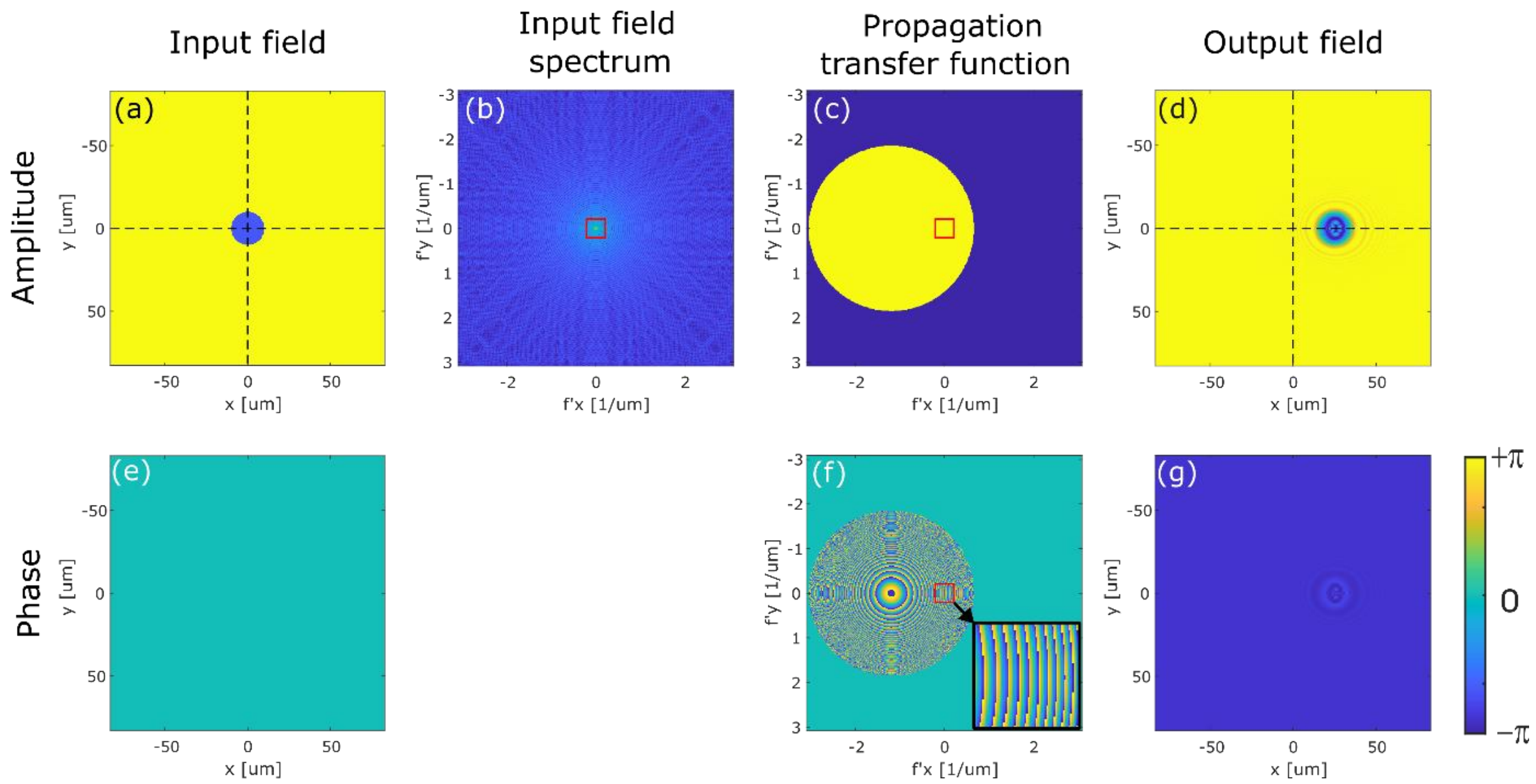

Fig. S4. Visualization of the modified Shift-Preserving, Tilt-Removed Angular Spectrum (SP-TR-AS) algorithm: amplitude (top row) and phase (bottom row) of ((a), (e)) the input optical field, (b) the spectrum of the input optical field, ((c), (f)) the propagation transfer function, and ((d), (g)) the output optical field. Results obtained for: wavelength $\lambda = 0.54$ μm, illumination angle $\alpha_x = 40°$, propagation distance $z = 30$ μm. The red rectangle indicates the Fourier frequency range that is transferred by lensless microscope with a pixel size of 2.4 μm. In SP-TR-AS, the tilt of the plane wave carrier is numerically removed, which supresses the boundary errors and enables using course sampling of the input signal.

The modified algorithm addresses the problem of border errors and ensures that the spectral region of interest is aligned with the centre of the computational matrix, allowing propagation to be performed with fewer computational resources (that is smaller spectral bandwidth and thus larger sampling interval Δ of the signal). However, one challenge remains. As shown in Figure S4(f), the phase of the propagation transfer function varies rapidly within the central region (region of interest marked with red frame). This means that to properly sample the transfer function, a high sampling frequency of the spectrum is required. A sampling interval of the spectrum is given by $\Delta_{fx} = 1/N/\Delta$, where N is the number of pixels. Therefore, to properly sample the transfer function, a large field of view is necessary. It is important to stress that this effect becomes very significant over large propagation distances (e.g., millimetre-scale backpropagation during hologram reconstruction in lensless microscopy), because the curvature of the propagation transfer function's phase distribution, $2\pi z\sqrt{\left(\frac{n_i}{\lambda}\right)^2 - f_x'^2 - f_y'^2}$, is inversely proportional to the propagation distance. This makes aliasing-free sampling of the propagation transfer function particularly challenging.

The described problem can be overcome by removing the phase tilt from the transfer function. The required tilt value is strictly related to the illumination vector and happens to be equal to transverse object wave shift vector (x_i, y_i) that results from the tilted propagation direction. This shift can be calculated from the geometry of the propagation task. To this aim, it is helpful to change Cartesian representation of the propagation angle α_x, α_y to spherical coordinates:

$$\alpha = \cos^{-1}\left(\frac{f_{iz}}{\frac{n}{\lambda}}\right) = \cos^{-1}\left(\frac{\sqrt{\left(\frac{n_i}{\lambda}\right)^2 - f_x^2 - f_y^2}}{\frac{n_i}{\lambda}}\right), \tag{S13}$$

$$\theta = \tan^{-1}\left(\frac{f_y}{f_x}\right), \tag{S14}$$

where α is a tilt with respect to the optical axis and θ is azimuth of illumination. The total shift in a transverse direction after propagation on a distance z is given by:

$$r = \mathrm{z} \cdot \tan\alpha. \tag{S15}$$

The transverse shift vector can be calculated from:

$$\begin{aligned} x_i &= r\cos\theta \\ y_i &= r\sin\theta \end{aligned}. \tag{S16}$$

The final AS-based propagation algorithm that is dedicated for the resources-efficient object wave backpropagation in lensless holotomography is described with Equations S8-S12, where Eq. (S10) is replaced with the following formula:

$$\widetilde{U}_z(f_x', f_y') = \widetilde{U}_{z=0}(f_x', f_y') exp\left[i2\pi z\sqrt{\left(\frac{n_i}{\lambda}\right)^2 - {f_x'}^2 - {f_y'}^2}\right] \exp\left[-i2\pi\left(f_x x_i + f_y y_i\right)\right]. \tag{S17}$$

Notably, this solution has already been proposed[S9] and applied for LHT[S10]. However, here we fully justify when and why this choice is optimal for LHT and support this claim with simulation-based analysis.

Removing the linear phase component of the transfer function [Eq. (S17)] reduces the sampling requirements of the signal (see slowly changing transfer function phase in Fig. S5(f)). Therefore, the challenging off-axis long-distance propagation can be accurately performed using minimal sampling, i.e., evaluation in shifted spectral coordinates enables relatively large pixel size that is typical for lensless microscopy, whereas the transfer function phase tilt removal enables using relatively small size of the computational matrix (number of pixels *N*). Additionality, in accordance with the Fourier shift theorem, the phase tilt removal in the Fourier domain results in the propagated optical field being represented in a shifted spatial coordinate system $(x - x_i,\ y - y_i)$. As a result, despite the tilted beam propagation, the diffraction shadow of the object occupies the same area of the computational matrix (compare position of the circle in the source (Fig. S5(a)) and destination plane (S5(d))). Therefore, removing the phase tilt not only enables optimal sampling conditions for propagation but also eliminates the sample's transverse shift, simplifying the data preprocessing pipeline for lensless holotomography.

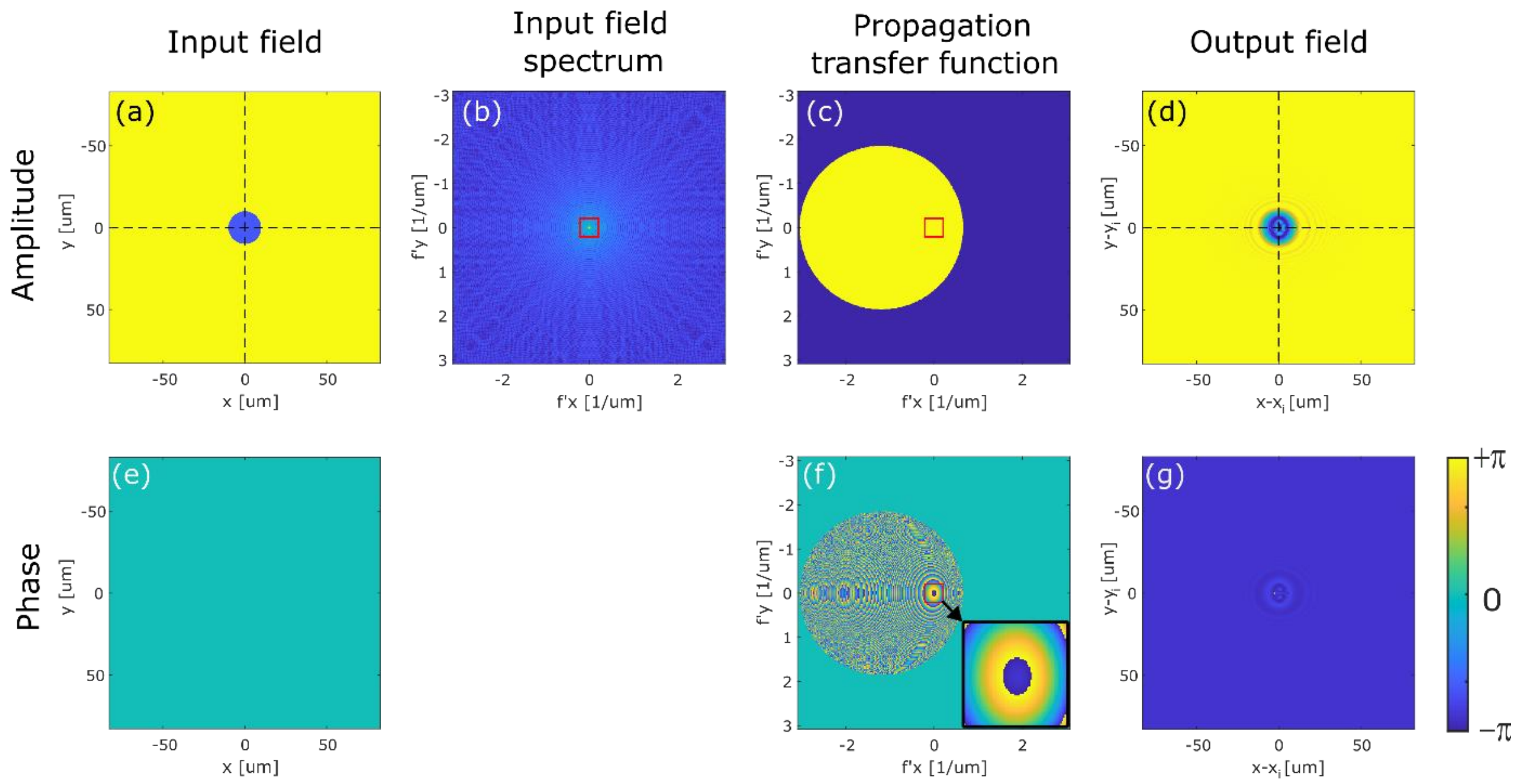

Fig. S5. Visualization of the modified Shift-Suppressed, Tilt-Removed Angular Spectrum (SS-TR-AS): amplitude (top row) and phase (bottom row) of ((a), (e)) the input optical field, (b) the spectrum of the input optical field, ((c), (f)) the propagation transfer function, and ((d), (g)) the output optical field. Results obtained for: wavelength $\lambda = 0.54\ \mu m$, illumination angle $\alpha_x = 40°$, propagation distance $z = 30\ \mu m$. The red rectangle indicates the Fourier frequency range that is transferred by lensless microscope with a pixel size of $2.4\ \mu m$. SS-TR-AS removes the carrier tilt and also numerically suppresses the associated transverse shift. This allows for a more compact computational domain (courser sampling and smaller field of view required).

In summary, this section describes two modified versions of the Angular Spectrum (AS) algorithm, tailored for the numerical modelling of the propagation of tilted optical fields. The first method, defined by equations S8–S12, is an angular spectrum propagation technique in which the tilt of the plane wave carrier is numerically removed, while the resulting transverse shift of the optical field is preserved. We refer to this method as Shift-Preserving, Tilt-Removed Angular Spectrum (SP-TR-AS). This approach mitigates boundary errors and enables propagation with coarser sampling of the computational grid.

The second method, defined by equations in order S8, S9, S13-S17, S11, also removes the carrier tilt but additionally suppresses the resulting transverse shift. This allows for a more compact computational domain and improves sampling efficiency. In particular, it enables accurate and aliasing-free long-distance propagation using coarse sampling and a small field of view. We refer to this method as Shift-Suppressed, Tilt-Removed Angular Spectrum (SS-TR-AS).

In the proposed LHT reconstruction scheme we employed both SP-TR-AS and SS-TR-AS propagations. SP-TR-AS method was used for propagation on short (i.e., $z = \Delta$, used in SOLVE) distances within the reconstructed volume, as the preservation of the transversal shift during propagation is necessary for volumetric reconstruction, and the short propagation distance does not introduce aliasing issues. SS-TR-AS method was employed for challenging off-axis propagation on larger distances (between camera and sample volume), which facilitated proper sampling of the propagation transfer function. Notably, the applied data processing pipeline—including data calibration, phase retrieval, and the tomographic reconstruction

algorithm—assumes working with a low-frequency-shifted field representation (with the carrier plane wave related to the tilted illumination removed). Therefore, we omitted steps 8 and 12 in our calculations.

Supplementary note 4: Multi-wavelength oblique illumination Gerchberg-Saxton phase retrieval for LHT data

Algorithm S1 implements a novel, computationally efficient multi-wavelength variant of the Gerchberg-Saxton algorithm. It is specifically designed for three-dimensional reconstruction under oblique illumination conditions, incorporating the captured intensities constraints and Ewald's sphere theory. The algorithm takes as input two intensity images, acquired with λ_1 and λ_2 wavelength for n^{th} illumination angle $(\alpha_{x_n}, \alpha_{y_n})$. It begins with initial guess, that the complex field at camera plane for first wavelength ($C_{\lambda_1 n}$) is equal to the square root of collected intensity image $I_{\lambda_1 n}$. Next, the iterative procedure begins, in which the complex field $C_{\lambda_1 n}$ is transformed to the $C_{\lambda_2 n}$ space, next the obtained $C_{\lambda_2 n}$ field is updated with collected $I_{\lambda_2 n}$ image and transformed back to $C_{\lambda_1 n}$ space. Iteration finishes with the $C_{\lambda_1 n}$ update with $I_{\lambda_1 n}$ image.

The mentioned above transform between general $U_{\lambda_1, z=0}$ and $U_{\lambda_2, z=0}$ fields is described with following steps:

(1) Fourier transform $U_{\lambda_1, z=0}$ according to Eq. (S9), which results in $\widetilde{U}_{\lambda_1, z=0}$,
(2) Multiply $\widetilde{U}_{\lambda_1, z=0}$ with the SS-TF-AS transfer function (Eq. (S15)) to propagate at $+z_{co}$ distance to the sample plan: $\widetilde{U}_{\lambda_1, z_{co}}$,
(3) Remaining in the Fourier domain, transfer to λ_2 domain using the Ewald sphere theory, which results in $\widetilde{U}_{\lambda_2, z_{co}}$,
(4) Multiply $\widetilde{U}_{\lambda_2, z_{co}}$ with the SS-TF-AS transfer function (Eq. (S15)) to propagate at $-z_{co}$ distance: $\widetilde{U}_{\lambda_2, z=0}$,
(5) Inverse Fourier transform of $\widetilde{U}_{\lambda_2, z=0}$ (Eq. (S8)): $U_{\lambda_2, z=0}$.

It is important to note that the employed cross-wavelength transformation method offers significant computational saving as it requires only two Fourier transforms (step 1 and 5) for each iteration. Notably, conventional multi-wavelength GS approaches[S11-S13] applies four Fourier transformations for each iteration – two Fourier transforms for propagation to the sample plane, in which the wavelength rescaling is achieved in space domain by direct multiplication of phase by the wavelength ratio $\varphi_{\lambda 2} = \varphi_{\lambda 1} \frac{\lambda_1}{\lambda_2}$, and then two Fourier transforms to propagate back to the camera plane. In our method, the wavelength rescaling is performed entirely in the Fourier domain, which significantly improves computational efficiency. As a result, the total computational cost is only two Fourier transforms plus the computation of two propagation kernels and one wavelength rescaling kernel (step 3, Eq. (S21) - explained below), which can be precomputed at the start of the GS algorithm.

Step (3) of our method is based on Wolf's theory[S14], which forms the theoretical foundation for tomographic reconstruction in holotomography and thus accounts for thick character of the sample. According to Wolf's theory, the optical field scattered by a 3D sample encodes information about the Fourier components of the sample's 3D scattering potential spectrum, $\tilde{O}$, which lie on a spherical cap. The radius of this cap is determined by the ratio of the surrounding refractive index to the wavelength of light, $\frac{n_i}{\lambda_{1,2}}$, and its position depends on the illumination direction. Specifically, the cap intersects the origin of the coordinate system and is oriented normal to the illumination direction.

In our GS method, we record two in-line holograms at different wavelengths for each illumination direction. Consequently, each angular projection corresponds to a pair of Ewald caps with different radii that intersect at the origin and are tangent to each other at that point, both oriented orthogonally to the illumination direction. This is illustrated in Fig. S6, which shows 9 pairs of Ewald caps corresponding to 9 illumination directions evenly distributed over a $\pm 40°$ angular range and two wavelengths (530 nm and 630 nm, denoted by green and red lines, respectively). As seen in Fig. S6, due to the relatively narrow range of transverse Fourier frequencies—a characteristic of lensless holographic tomography (HT) compared to lens-based HT—the two Ewald caps are closely spaced. In our method, we neglect this slight spatial offset and assume that the Ewald caps occupy the same volume. Under this approximation, we derive a quantitative relationship between the two in-line holograms, since both capture information from the same region of the scattering potential. This enables us to define a transformation between the two wavelength domains.

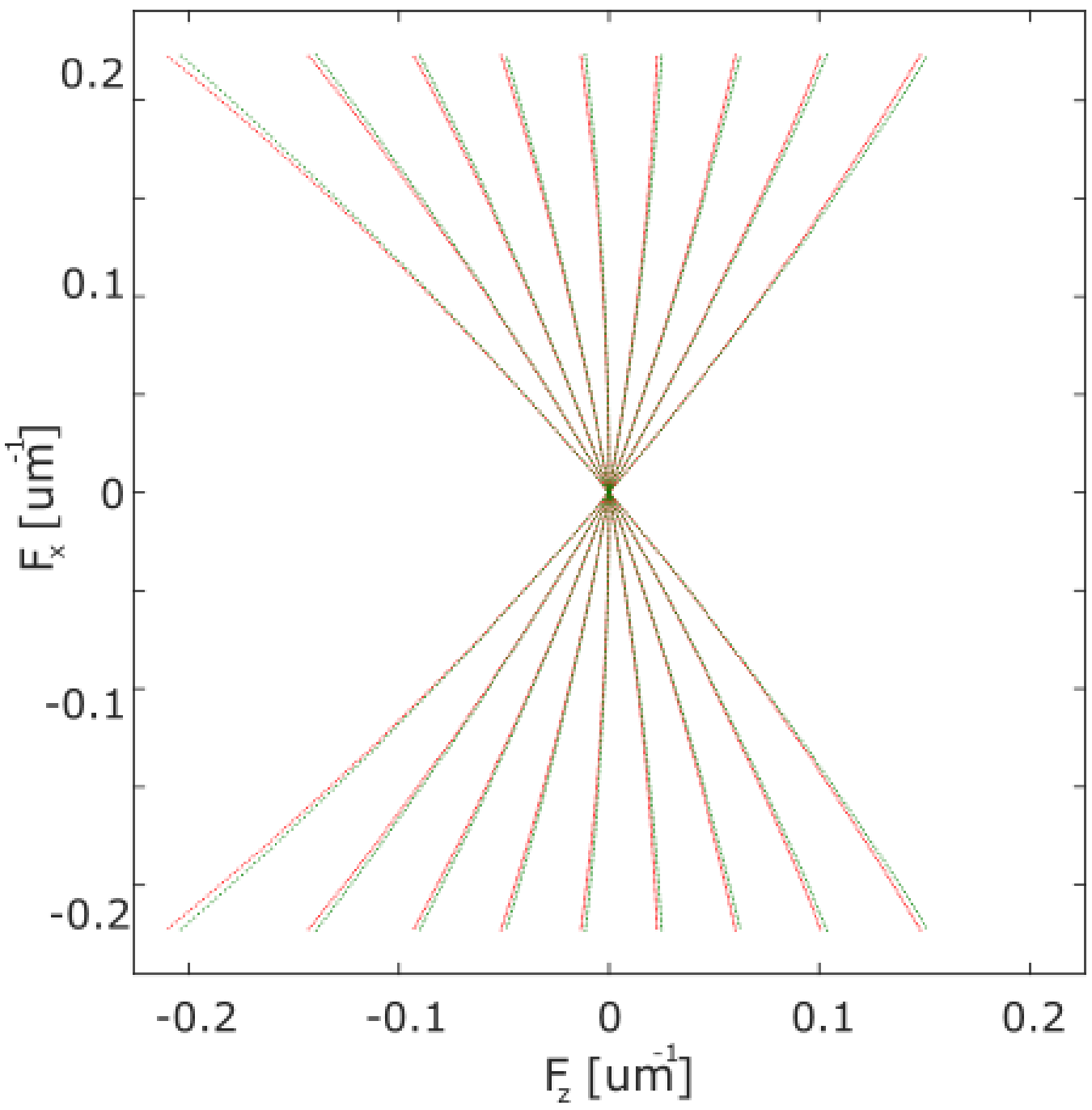

Fig. S6. Ewald spheres corresponding to two wavelengths (530 nm and 630 nm for red and green lines, respectively) and 9 illumination angles uniformly distributed over a $\pm 40°$ angular range.

In order to establish this transform, let us first recall the first-order Born approximation from Wolf's theory, which defines the quantitative relationship between the scattered optical field and the Fourier spectrum of the scattering potential:

$$\widetilde{U}_{\lambda,z_{co}}(F_x, F_y) = \frac{\left(\frac{2\pi}{\lambda}\right)^2}{2i2\pi f_z} \tilde{O}(F_x, F_y, F_z). \tag{S18}$$

In the above formula (F_x, F_y, F_z) are Fourier coordinates of the 3D spectrum. The transverse coordinates are related to the spatial frequencies of the optical fields by: $F_x = f_x - f_{ix} = f_x'$, $F_y = f_y - f_{iy} = f_y'$, where the illumination vector $f_{ix} = \frac{n_i}{\lambda}\sin\alpha_x$, $f_{iy} = \frac{n_i}{\lambda}\sin\alpha_y$ (see Sec. Tailored angular spectrum propagation algorithm for lensless holotomography). The axial Fourier coordinates are defined by $F_z = f_z - f_{iz}$, where $f_z = \sqrt{\left(\frac{n_i}{\lambda}\right)^2 - {f_x}^2 - {f_y}^2}$ and $f_{\mathrm{iz}} = \sqrt{\left(\frac{n_i}{\lambda}\right)^2 - f_{ix}^2 - f_{iy}^2}$. Now, if we disregard the difference in location of Ewald caps due to change in light wavelength, we can claim that:

$$\frac{\left(\frac{2\pi}{\lambda_1}\right)^2}{2i2\pi f_z(\lambda_1)} \cdot \widetilde{U}_{\lambda 2,z_{co}} = \frac{\left(\frac{2\pi}{\lambda_2}\right)^2}{2i2\pi f_z(\lambda_2)} \cdot \widetilde{U}_{\lambda 1,z_{co}}\,, \tag{S19}$$

which, after simplification, provides the cross-wavelength transform equation:

$$\widetilde{U}_{\lambda 2,z_{co}} = \left(\frac{\lambda_1}{\lambda_2}\right)^2 \frac{f_z(\lambda_1)}{f_z(\lambda_2)} \cdot \widetilde{U}_{\lambda 1,z_{co}}\,, \tag{S20}$$

which can be computed as:

$$\widetilde{U}_{\lambda_2,z_{co}} = \left(\frac{\lambda_1}{\lambda_2}\right)^2 \frac{\sqrt{\left(\frac{n_i}{\lambda_1}\right)^2 - \left(f_x' + \frac{n_i}{\lambda_1} sin\alpha_x\right)^2 - \left(f_y' + \frac{n_i}{\lambda_1} sin\alpha_x\right)^2}}{\sqrt{\left(\frac{n_i}{\lambda_2}\right)^2 - \left(f_x' + \frac{n_i}{\lambda_2} sin\alpha_x\right)^2 - \left(f_y' + \frac{n_i}{\lambda_2} sin\alpha_x\right)^2}} \cdot \widetilde{U}_{\lambda_1,z_{co}}\,, \tag{S21}$$

where (f_x', f_y') denote the spatial frequency coordinates resulting from the Fast Fourier Transform of optical fields $U_{\lambda_{1,2},z_{co}}$ (it is assumed that $U_{\lambda_{1,2},z_{co}}$ has removed phase tilt related to inclined illumination, see explanation in supplementary note 3).

Algorithm S1 – multi-wavelength Gerchberg-Saxton (GS)

Inputs: $I_{\lambda_1 n}(x,y), I_{\lambda_2 n}(x,y), \alpha_{x_n}, \alpha_{y_n}, \lambda_1, \lambda_2, z_{co}, N_{iter}$
Output: $C_{\lambda_1 n}(x,y)$

$C_{\lambda_1 n}(x,y) = \sqrt{I_{\lambda_1 n}(x,y)}$ % Initial guess
for: $n_i = 1 : N_{iter}$
  $C_{\lambda_2 n}(x,y) = cross\_wavelength\_transform\{C_{\lambda_1 n}(x,y), z_{co}, \lambda_1, \lambda_2, \alpha_{x_n}, \alpha_{y_n}\}$
  $C_{\lambda_2 n}(x,y) = \frac{C_{\lambda_2 n}(x,y)}{|C_{\lambda_2 n}(x,y)|}\sqrt{I_{\lambda_2 n}(x,y)}$ % Update with collected image
  $C_{\lambda_1 n}(x,y) = cross\_wavelength\_transform\{C_{\lambda_2 n}(x,y), z_{co}, \lambda_2, \lambda_1, \alpha_{x_n}, \alpha_{y_n}\}$
  $C_{\lambda_1 n}(x,y) = \frac{C_{\lambda_1 n}(x,y)}{|C_{\lambda_1 n}(x,y)|}\sqrt{I_{\lambda_1 n}(x,y)}$ % Update with collected image

Supplementary note 5: Filtered backpropagation LHT reconstruction

Algorithm S2 present the implementation of the filtered backpropagation (FB) algorithm. The algorithm takes as input a set of N complex optical fields $C(x,y)$ acquired for different illumination angles α_x, α_y and a λ_1 wavelength, along with measured volume axial sampling Δz and a user specified range of propagation distances z_{min}, z_{max} that correspond to minimal and maximal axial distance between sample volume and camera. The algorithm relies on propagating each nth optical field: firstly at $-z_{co}$ distance with SS-TR-AS method (more accurate for longer propagation distances) and then within z_{min}, z_{max} range with SP-TR-AS method (necessary to account for transversal shift for volume reconstruction). For each given z, all N propagated fields are averaged to produce the x, y slice of the final $U(x,y,z)$ volume.

**Algorithm S2 – filtered backpropagation (FB) method**
**Inputs:** $C(x,y), \alpha_x, \alpha_y, \lambda_1, z_{min}, z_{max}, \Delta z$ **Outputs:** $U(x,y,z)$
$U = 0$
**for:** $n = 1: N$
$U_{z_{co}}(x,y) = \text{SS-TR-AS}\{C_n(x,y), -z_{co}, \alpha_{x_n}, \alpha_{y_n}, \lambda_1\}$
**for:** $z = z_{min}: \Delta z: z_{max}$
$U(x,y,z) = U(x,y,z) + \text{SP-TR-AS}\{U_{z_{co}}(x,y), (z_{co} - z), \alpha_{x_n}, \alpha_{y_n}, \lambda_1\}$
$U = U/N$

Supplementary note 6: SOLVE algorithm description

Proposed SOLVE algorithm is described in detail via pseudo-code presented in Algorithm S3. Firstly, an initial estimate of the 3D complex amplitude distribution is obtained using a modified filtered backpropagation (FB) algorithm (Algorithm S2 in Supplementary Document 1) for the user defined range of propagation distances (z_{min} and z_{max} corresponding to minimal and maximal distance between sample volume and camera) and the measured volume axial sampling Δ_z. The final 3D distribution U, is then refined using the proposed iterative reconstruction algorithm.

Each iteration of the SOLVE algorithm begins with filtering the 3D complex volume U by applying a physical constraint that the normalized object amplitude must be less than or equal to 1 – meaning the object can absorb but not generate light. This step improves the convergence and stability of the reconstruction process. Other constraints could also be applied, such as total variation denoising or assuming that the object's refractive index is either greater or smaller than that of the surrounding medium. Next, for each illumination angle n, the multi-plane volume U is propagated to the camera plane using a multi-slice beam propagation method (BPM) model[S15, S16]. The propagation between individual slices of U is realized with the use of specialized off-axis shift-preserving AS method (SP-TR-AS), while propagation between last slice of U and camera is realized with shift-suppressed AS (SS-TR-AS). Both methods were originally adopted by us to lensless large volume reconstruction with

oblique illumination regime. Alternatively to previous works in this context offering general views, we provide scrupulous analysis and rigorous justification for the algorithm steps and their implementation within lensless regime. The difference ΔC_n between the estimated field at the camera (C_n') and the measured complex amplitude C_n (retrieved from GS) is then calculated. After computing the set of differences ΔC_1 to ΔC_N, a 3D correction volume ΔU is reconstructed using the FB method[S17]. This ΔU is subtracted from the current U with a learning rate ε, resulting in the updated estimate.

The parameter ε is selected empirically by observing reconstruction quality over subsequent iterations: too large ε value leads to oscillations in the background phase, while too small ε value results in minimal improvement. In this study, ε was empirically set between 0.005 and 0.025, depending on the dataset, which is generally a recommendable range. The SOLVE algorithm was typically run for five iterations, as no significant improvement was usually achieved within that range.

Algorithm S3 – SOLVE

Inputs: $C(x,y), \alpha_x, \alpha_y, \lambda_1\ z_{min}, z_{max}, \Delta_z, N_{iter}, \epsilon$
Outputs: $U(x,y,z)$

% Initial guess (FB)
$U(x,y,z) = \mathrm{FB}(C(x,y), \alpha_x, \alpha_y, \lambda_1, z_{min}, z_{max}, \Delta_z)$
% Iterative reconstruction
for: $n_i = 1{:}\,N_{iter}$
  % Absorption constraint
  $A(x,y,z) = |U(x,y,z)|$
  $A(A > 1) = 1$
  $U(x,y,z) = A(x,y,z) \cdot \frac{U(x,y,z)}{|U(x,y,z)|}$
  % Forward propagation (multi-slice BPM)
  for: $n = 1{:}\,N$
    $U_z(x,y) = U(x,y,z_{max})$
    for: $z = z_{max}{:} -\Delta_z{:}\, z_{min}$
      % Propagate to next plane
      $U_{z-\Delta z}(x,y) = \text{SP-TR-AS}(U_z(x,y), -\Delta_z, \alpha_{x_n}, \alpha_{y_n}, \lambda_1)$
      % Actualize field with next field plane
      $U_{z-\Delta_z}(x,y) = U_{z-\Delta_z}(x,y) \cdot U(x,y,z-\Delta_z)$
    % Propagate to camera plane
    $C_n'(x,y) = \text{SS-TR-AS}(U_z(x,y), -z_{min}, \alpha_{x_n}, \alpha_{y_n}, \lambda_1)$
    % Calculate difference between estimation and measurement
    $\Delta C_n(x,y) = C_n'(x,y) - C_n(x,y)$
  % 4. Backward propagation (FB)
  $\Delta U(x,y,z) = \mathrm{FB}(\Delta C(x,y), \alpha_x, \alpha_y, \lambda_1, z_{min}, z_{max}, \Delta_z)$
  % 5. Update U
  $U(x,y,z) = U(x,y,z) - \varepsilon \cdot \Delta U(x,y,z)$

Supplementary note 7: SOLVE's robustness to cut projections

In lensless holotomography, data acquisition takes place under defocused conditions. When combined with tilted illumination, this results in a variable transverse shift of the object projections. One consequence of this transverse projection shift is the possibility of extending FOV. As the azimuth of the illumination beam changes, the detector captures information from different regions of the object, leading to a potential increase of FOV by $2 \cdot z_{co} \cdot \tan\alpha$, where z_{co} is the distance between the sample plane and the detector, and α denotes the illumination angle (tilt of the illumination beam with respect to the optical axis). For typical parameters used in our system, $z_{co} = 1.72$ mm and $\alpha = 42°$, this gives a potential FOV enlargement of 3.1 mm.

Conversely, the transverse projection shift may have an adverse effect, whereby some object information may fall outside the detector area, as observed in the mouse brain slice experiment shown in Fig. 5 of the main manuscript. Despite this truncation, the reconstruction quality remained largely unaffected. To better understand this effect, let us apply 3D transfer function formalism. Figs. S7(a), S7(b) show a pair of 3D views of the volumetric transfer function for lensless holotomography in the case of a complete tomographic data set (full azimuthal coverage). The transfer function was obtained for conical illumination at an incidence angle of $40°$, a wavelength of $\lambda = 0.54$ µm, and a surrounding medium refractive index of $n_i = 1$. The transverse resolution is limited by the pixel size, $\Delta_x = 2.4$ µm.

The upper edge of the mouse brain slice sample presented in Fig. 5(a) of the manuscript fell outside the camera field of view in approximately 30% of the projections (i.e., for azimuth angles from $216°$ to $324°$). We generated the transfer function for this condition (Figs. S7(c), S7(d)). It can be observed that the loss of information is relatively small. Specifically, the volume of the transferred object spectrum decreases from 0.0445 μm^{-3} for the full projection set to 0.0431 μm^{-3}, corresponding to only a 3% reduction. We also investigated the worsen case in which half of the projections are missing (Fig. S7 (e), (f)). In this case, the volume of the transferred object spectrum is 0.0369 μm^{-3}, which represents a 17% decrease.

These results demonstrate that while LHT acquisition conditions inherently produce transverse projection shifts—potentially enlarging the FOV but also causing projection truncation—the system is robust to these effects. The 3D transfer function analysis confirms that even significant truncation leads to only modest reductions in the reconstructed object spectrum, highlighting the resilience of LHT under limited-angle or partially truncated acquisition scenarios.

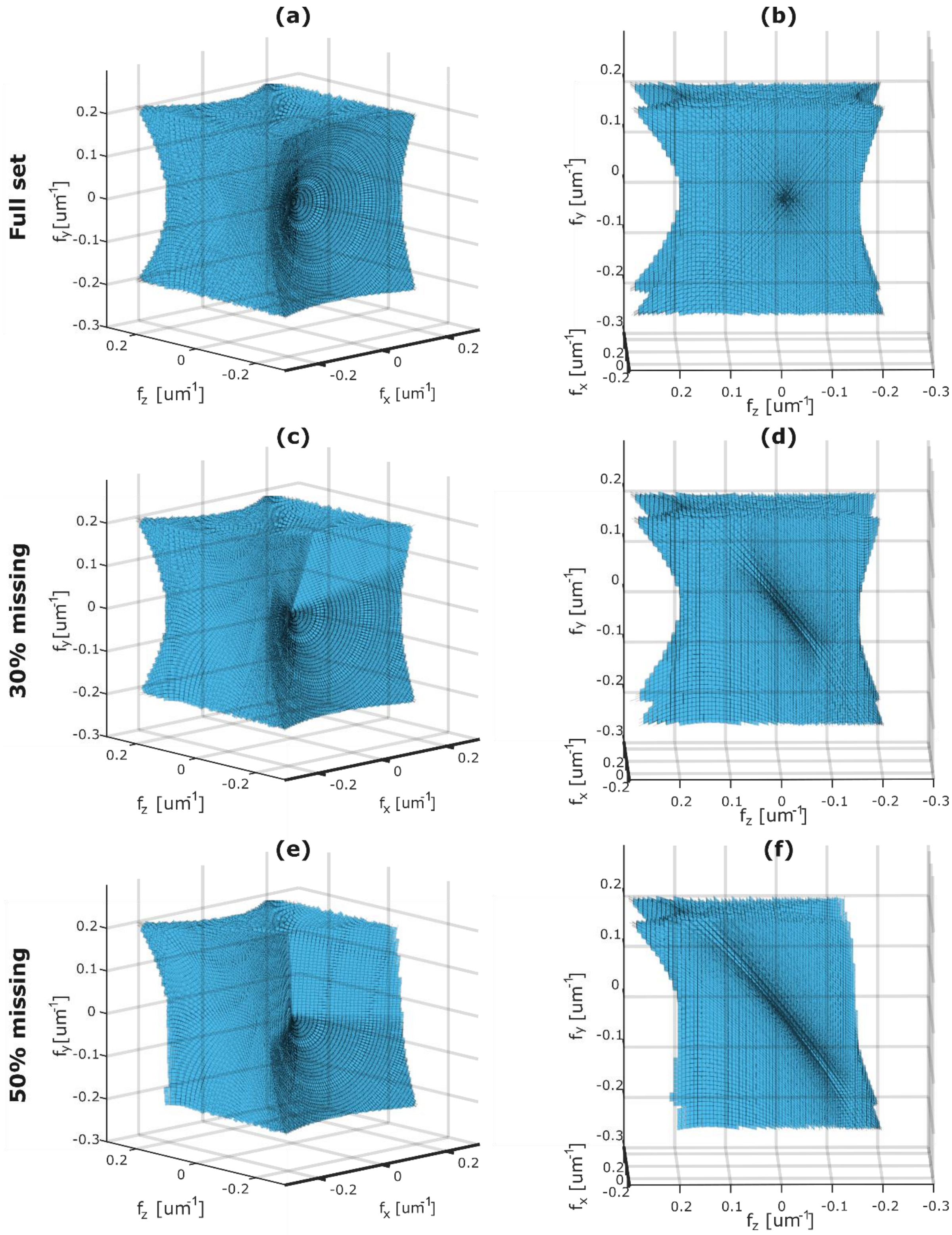

Fig. S7. Pairs of different 3D renderings of the transfer function for LHT systems with ((a), (b)) full azimuthal coverage, ((c), (d)) 30% missing projections, and ((e), (f)) 50% missing projections. The analysis assumes conical illumination at an incidence angle of $40°$, a wavelength of $\lambda = 0.54$ μm, and a surrounding medium refractive index of $n_i = 1$. The transverse resolution is limited by the pixel size, $\Delta_x = 2.4$ μm.

Supplementary note 8: SOLVE algorithm implementation, optimization and reconstruction times

The main computational cost of the SOLVE algorithm arises from two core modules: the FB module (lines 1 and 13 in Algorithm S3) and the BPM module (lines 7–10 in Algorithm S3). In each SOLVE iteration, the FB module is executed once, whereas the BPM module is executed independently for each acquired projection (in our case $N = 180$). Both modules were optimized individually and ported to GPU execution to reduce reconstruction time.

The FB module implementation follows Algorithm S2, but was optimized by eliminating the time-consuming "for" loop in line 3 with parallel calculations. Specifically, for each nth projection, a 3D set of 2D propagation kernels (corresponding to the exponential term in Eq. (S10)) is generated simultaneously for all reconstructed axial positions. The size of this kernel set matches the dimensions of the reconstructed volume ($S_x \times S_y \times S_z$). The two-dimensional Fourier transform of the field $U_{z_{co}}$ is then multiplied with all kernels in parallel, followed by an inverse two-dimensional Fourier transform, yielding a full 3D reconstruction for a given projection in a single operation.

The BPM module was implemented according to Algorithm S3 (lines 7–11). To reduce computational overhead, the SP-TR-AS propagation kernel (line 9) was precomputed prior to the "for" loop in line 8, as it remains constant for all axial propagation steps associated with a given illumination angle.

The BPM module operates on two-dimensional arrays of size $S_x \times S_y$, resulting in relatively modest memory requirements (e.g., a 4000 × 4000 array occupies approximately 128 MB), which allows execution even on mid-range GPUs. In contrast, the FB module operates on large three-dimensional volumes (e.g., a 4000 × 4000 × 400 array requires approximately 51.2 GB of memory), necessitating the use of high-end GPU hardware. Consequently, for extremely large volumes – such as the brain slice dataset presented in the manuscript – the FB module was executed without GPU acceleration.

Table S1 summarizes the processing times for different reconstructed volumes and for the FB, BPM, and full SOLVE pipelines. In all cases, the SOLVE algorithm was run for $N_{iter} = 5$ iterations, corresponding to $N_{iter} + 1 = 6$ executions of the FB module and $N_{iter} \cdot N = 5 \cdot 180 = 900$ executions of the BPM module. All benchmarks were performed on a workstation equipped with an NVIDIA RTX 6000 Ada Generation GPU and an Intel Xeon w9-3475X processor.

Table S1. Processing tam of FB, BPM and SOLVE methods for different sizes of reconstructed volumes.

$S_x \times S_y \times S_z$	$400 \times 400 \times 400$	$100 \times 400 \times 400$	$400 \times 400 \times 100$	$800 \times 800 \times 100$	$1600 \times 1600 \times 100$
**FB CPU [s]**	157.16	44.68	38.46	157.98	601.97
**FB GPU [s]**	2.95	1.22	1.05	3.37	17.17
**BPM GPU [s]**	0.24	0.13	0.05	0.23	1.08
**SOLVE (FB CPU + BPM GPU) [s]**	1129.49	329.46	317.20	1167.82	4295.28
**SOLVE GPU [s]**	200.33	66.59	81.99	236.05	763.81

References

S1. https://www.printables.com/model/217557-coliseum

S2. T. Kozacki and K. Falaggis, "Angular spectrum-based wave-propagation method with compact space bandwidth for large propagation distances," Opt. Lett. **40**, 3420-3423 (2015).

S3. T. Kozacki and K. Falaggis, "Angular spectrum method with compact space–bandwidth: generalization and full-field accuracy," Appl. Opt. **55**, 5014-5024 (2016).

S4. A. F. Müller, R. B. Bergmann, Claas Falldorf, "High resolution lensless microscopy based on Fresnel propagation," Opt. Eng. **63**(11) 111805 (2024).

S5. J. W. Goodman, *Introduction to Fourier Optics*, 3rd ed. (Roberts and Company Publishers, 2005).

S6. J. Kostencka, T. Kozacki, A. Kuś, B. Kemper, and M. Kujawińska, "Holographic tomography with scanning of illumination: space-domain reconstruction for spatially invariant accuracy," *Biomed. Opt. Express* **7**, 4086–4101 (2016).

S7. T. -a. Pham, E. Soubies, A. Ayoub, J. Lim, D. Psaltis and M. Unser, "Three-Dimensional Optical Diffraction Tomography With Lippmann-Schwinger Model," in *IEEE Transactions on* Computational Imaging **6**, 727-738 (2020).

S8. F. Soulez, "Gauging diffraction patterns: field of view and bandwidth estimation in lensless holography," Appl. Opt. **60**, B38-B48 (2021).

S9. C.-S. Guo, Y.-Y. Xie, and B. Sha, "Diffraction algorithm suitable for both near and far field with shifted destination window and oblique illumination," Opt. Lett. 39(8), 2338–2341 (2014).

S10. Z.-W. Qin, Y. Yang, Y.-L. Ma, Y.-B. Han, X.-L. Liu, H.-Y. Huang, C.-S. Guo, and Q.-Y. Yue, "Portable single-shot lens-free tomographic microscope for imaging dynamic specimens," *Opt. Express* **32**, 29329–29343 (2024).

S11. C. Zuo, J. Sun, J. Zhang, Y. Hu, and Q. Chen, "Lensless phase microscopy and diffraction tomography with multi-angle and multi-wavelength illuminations using a LED matrix," *Opt. Express* **23**, 14314-14328 (2015).

S12. P. Bao, G. Pedrini, and W. Osten, “Optical surface profile measurement using phase retrieval by tuning the illumination wavelength,” *Opt. Commun.* **285**, 5029-5036 (2012).

S13. V. Micó, M. Rogalski, J.Á. Picazo-Bueno, and M. Trusiak “Single-shot wavelength-multiplexed phase microscopy under Gabor regime in a regular microscope embodiment,” *Sci. Rep.* **13**, 4257 (2023).

S14. E. Wolf, “Three-dimensional structure determination of semi-transparent objects from holographic data,” *Opt. Commun.* **1**, 153–156 (1969).

S15. W. Pierré, L. Hervé, C. Paviolo, O. Mandula, V. Remondiere, S. Morales, S. Grudinin, P. F. Ray, M. Dhellemmes, C. Arnoult, and C. Allier, "3D time-lapse imaging of a mouse embryo using intensity diffraction tomography embedded inside a deep learning framework," Appl. Opt. **61**, 3337-3348 (2022).

S16. J. Kim, B. Bolton, K. Moshksayan, R. Khanna, M. E. Swartz, M. Ziemczonok, M. Kamra, K. A. Jorn, S. H. Parekh, M. Kujawińska, J. Eberhart, E. S. Cenik, A. Ben-Yakar, S. Chowdhury, “Inverse-scattering in biological samples via beam-propagation,” bioRxiv https://doi.org/10.1101/2025.08.17.670744.

S17. W. Zheng, J. Wang, Y. Zhou, Q. Zeng, C. Z., L. Liu, H. Yu, and Y. Yang, "Lensless holographic microscope with a time and memory-saving algorithm for large-volume imaging of organoids," Opt. Lett. 48, 771-774 (2023)
